# Analysis of the Clinical Pipeline of Treatments for Drug-Resistant Bacterial Infections: Despite Progress, More Action Is Needed

**DOI:** 10.1128/aac.01991-21

**Published:** 2022-03-15

**Authors:** Mark S. Butler, Valeria Gigante, Hatim Sati, Sarah Paulin, Laila Al-Sulaiman, John H. Rex, Prabhavathi Fernandes, Cesar A. Arias, Mical Paul, Guy E. Thwaites, Lloyd Czaplewski, Richard A. Alm, Christian Lienhardt, Melvin Spigelman, Lynn L. Silver, Norio Ohmagari, Roman Kozlov, Stephan Harbarth, Peter Beyer

**Affiliations:** a MSBChem Consulting, Brisbane, Queensland, Australia; b Institute for Molecular Bioscience, University of Queensland, Brisbane, Queensland, Australia; c Antimicrobial Resistance Division, WHO, Geneva, Switzerland; d F2G Limited, Eccles, Manchester, United Kingdom; e McGovern Medical School, The University of Texas Health Science Center at Houston, Houston, Texas, USA; f Scientific Advisory Committee, GARDP, Geneva, Switzerland; g The National Biodefense Science Board, U.S. Department of Health and Human Services, Washington, DC, USA; h Center for Infectious Diseases Research, Houston Methodist Research Institute, Houston, Texas, USA; i Center for Infectious Diseases, UTHealth School of Public Health, Houston, Texas, USA; j Infectious Diseases Institute, Rambam Health Care Campus, Haifa, Israel; k The Ruth and Bruce Rappaport Faculty of Medicine, Technion-Israel Institute of Technology, Haifa, Israel; l Oxford University Clinical Research Unit, Ho Chi Minh City, Vietnam; m Centre for Tropical Medicine and Global Health, Nuffield Department of Clinical Medicine, Oxford University, Oxford, United Kingdom; n Chemical Biology Ventures Ltd., Abingdon, Oxfordshire, United Kingdom; o CARB-X, Boston University, Boston, Massachusetts, USA; p Université de Montpellier, INSERM, Institut de Recherche pour le Développement, Montpellier, France; q TB Alliance, New York, New York, USA; r LL Silver Consulting, Springfield, New Jersey, USA; s National Center for Global Health and Medicine, Tokyo, Japan; t Institute of Antimicrobial Chemotherapy, Smolensk State Medical University, Smolensk, Russia; u National Center for Infection Prevention, Swissnoso, Bern, Switzerland; v Infection Control Programme, Geneva University Hospitals and Faculty of Medicine, WHO Collaborating Center for Patient Safety, Geneva, Switzerland

**Keywords:** antibacterial pipeline, antibiotic, traditional, nontraditional, clinical trials, WHO priority pathogens, tuberculosis, mycobacteria, *Clostridioides difficile*

## Abstract

There is an urgent global need for new strategies and drugs to control and treat multidrug-resistant bacterial infections. In 2017, the World Health Organization (WHO) released a list of 12 antibiotic-resistant priority pathogens and began to critically analyze the antibacterial clinical pipeline. This review analyzes “traditional” and “nontraditional” antibacterial agents and modulators in clinical development current on 30 June 2021 with activity against the WHO priority pathogens mycobacteria and Clostridioides difficile. Since 2017, 12 new antibacterial drugs have been approved globally, but only vaborbactam belongs to a new antibacterial class. Also innovative is the cephalosporin derivative cefiderocol, which incorporates an iron-chelating siderophore that facilitates Gram-negative bacteria cell entry. Overall, there were 76 antibacterial agents in clinical development (45 traditional and 31 nontraditional), with 28 in phase 1, 32 in phase 2, 12 in phase 3, and 4 under regulatory evaluation. Forty-one out of 76 (54%) targeted WHO priority pathogens, 16 (21%) were against mycobacteria, 15 (20%) were against C. difficile, and 4 (5%) were nontraditional agents with broad-spectrum effects. Nineteen of the 76 antibacterial agents have new pharmacophores, and 4 of these have new modes of actions not previously exploited by marketed antibacterial drugs. Despite there being 76 antibacterial clinical candidates, this analysis indicated that there were still relatively few clinically differentiated antibacterial agents in late-stage clinical development, especially against critical-priority pathogens. We believe that future antibacterial research and development (R&D) should focus on the development of innovative and clinically differentiated candidates that have clear and feasible progression pathways to the market.

## INTRODUCTION

**FIG 1:**
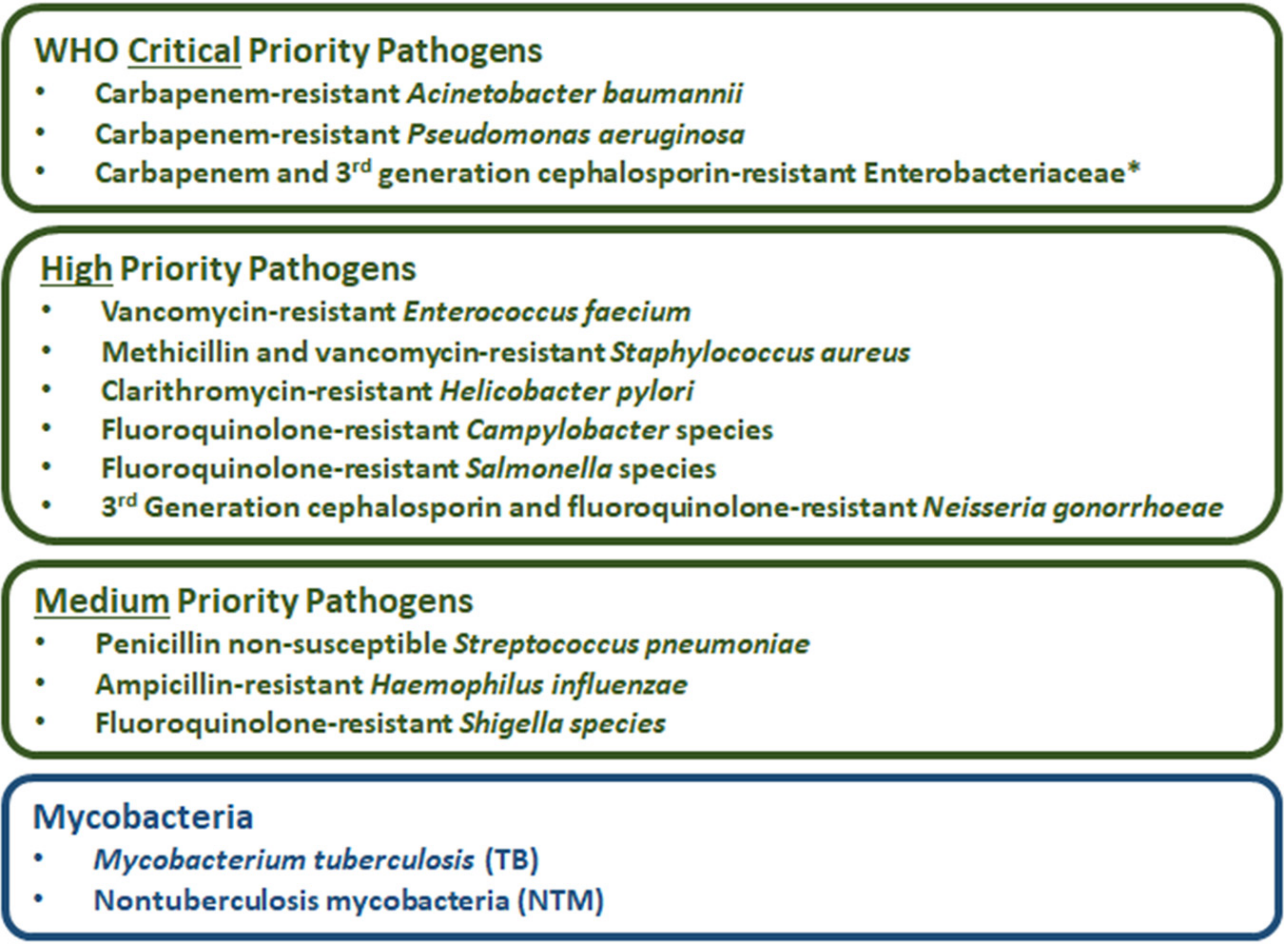
List of the WHO’s critical-, high-, and medium-priority pathogens (3, 4) and mycobacteria. *, *Enterobacteriaceae* (Escherichia coli, Enterobacter spp., and Klebsiella pneumoniae) and *Enterobacterales* (*Morganella* spp., Proteus spp., *Providencia* spp., and *Serratia* spp.).

The need for new antibacterial drugs to treat multidrug-resistant (MDR) bacterial infections is a critical global health issue, which has been recognized by many governmental, nongovernmental, and intergovernmental organizations (1, 2), including the World Health Organization (WHO). In February 2017, the WHO released a list of 12 antibiotic-resistant priority pathogens (Fig. 1), which are still among the most important bacterial infectious threats to human health (3–5). The WHO has also been critically analyzing the antibacterial pipeline since 2017, along with The Pew Charitable Trusts (6), which has resulted in the publication of four reports in 2017 (7), 2018 (8), 2019 (9), and 2021 (10). These pipeline reports and the WHO bacterial priority pathogen list have been used by policy makers, funders/sponsors, researchers, and developers to help guide the discovery and development of new antibacterial treatments. The U.S. Centers for Disease Control and Prevention (CDC) also released important pathogen (bacteria and fungi) threat lists in 2019 (11) and 2013 (12). While the WHO and CDC lists mostly overlap, there are some differences: the WHO list has ampicillin-resistant Haemophilus influenzae as a medium-priority pathogen, while the CDC list has Clostridioides difficile as an urgent threat, erythromycin-resistant group A Streptococcus and clindamycin-resistant group B Streptococcus as concerning threats, and drug-resistant Mycoplasma genitalium and Bordetella pertussis on a watch list. In March 2021, India released its own priority pathogen list (13), which included two pathogens, coagulase-negative staphylococci (CoNS) and Neisseria meningitidis (meningococcal disease), that are not in the WHO and CDC lists. The WHO is planning to update their priority pathogen list in 2022.

The discovery of new antibacterial drugs with activity against MDR bacteria is very challenging due to difficulties in designing products with suitable physicochemical properties (leading to desirable pharmacokinetics/pharmacodynamics properties) and acceptable toxicity profiles. Another major challenge is the lack of a suitable economic model that can provide long-term support for biotech and small companies developing new antibacterial agents (14–17). Factors underlying the lack of support include (i) the fact that antibacterial treatments are available for most bacterial infections, with most available as inexpensive generics, (ii) the typical short treatment duration of acute bacterial infections (18), (iii) the time and cost associated with traditional research and development (R&D) models, (iv) stewardship measures that—aiming at preserving new antibiotics efficacy—appropriately encourage prescribers to reserve new antibiotics and place them in the bottom of clinical guidelines as last-resort treatments, and (v) a lack of funding for phase 2 and 3 trials (19). All these elements have led to a market environment that is only marginally, if at all, profitable for most antibacterial drug developers. For example, the highest revenue for a patent protected antimicrobial in the United States in 2018 was US$138 million for the cephalosporin ceftaroline (17). The top 10 antimicrobials by sales in the United States in 2018, which included nine antibacterial drugs and the antifungal isavuconazole, had a total revenue of US$644 million. This drops down even further for the antibacterial drugs ranked 6 to 10 in sales (total sales revenues US$136 million, average $27.2 million). This is in stark contrast to the top-selling 2018 drug, adalimumab (therapeutic area: rheumatology), which had a total U.S. revenue of US$13.680 billion; even the revenue from the 10th highest selling drug in 2018, the anticoagulant apixaban, had US$3.76 billion revenue. This significant discrepancy in revenue helps to explain why most of the large pharmaceutical companies have either stopped or reduced their antibacterial R&D programs (19).

The WHO has published an economic model that demonstrates these financial challenges (20). To address this issue, several “push” and “pull” development incentives are being proposed and implemented in several countries (21–25). Push-funding policies aim to reduce early development costs of developers by providing funding (e.g., grant support, contract funding, tax incentives, and private/public partnerships), while pull-funding policies aim to optimize the late stage of drug development and create viable market demand for sponsors (e.g., market entry rewards, extended exclusivity period, tradable market voucher, and higher reimbursement) (26). For example, the United Kingdom’s antibiotic subscription pilot is the first ever fully delinked antibiotic pull incentive (27, 28). In the United States, the PASTEUR Act is a bipartisan bill that, if passed into law, would similarly create a delinked reward model for novel and clinically needed new antimicrobials (29, 30).

In the last few years, there has been an increase in so-called “nontraditional” approaches to antibacterial therapy, developing drugs that have different modes of action compared to the “traditional” direct-acting antibacterial agents (31, 32). These nontraditional antibacterial agents can prevent or treat bacterial infections through several modes of action, including directly or indirectly inhibiting bacterial growth, inhibiting virulence, ameliorating resistance, restoring the gut microbiome, or boosting the immune system to clear infections (Table 1). However, most of these candidates are being clinically evaluated (33) as adjuvant therapies in combination with “standard of care” antibiotics. To date, there have been only three nontraditional antibacterial agents approved, all of which are monoclonal antibodies (MAb). Bezlotoxumab (approved by the FDA in 2016) binds and neutralizes Clostridioides difficile toxin B and was approved after the completion of two phase 3 clinical trials (NCT01241552 and NCT01513239) (34). Raxibacumab was authorized for the treatment of inhalational anthrax in adults and children (approved 2012 by the FDA) (35). Obiltoxaximab (US FDA, 2016; EMA, 2020) (36), like raxibacumab, has been approved to control the symptoms of inhaled anthrax toxins; while the safety profiles of raxibacumab and obiltoxaximab have been investigated in healthy volunteers, fortunately they have not yet been used clinically (37).

**TABLE 1:** The five classification categories of nontraditional antibacterial agents

Nontraditional classification	Definition
Antibodies	A protein component of the immune system (or synthetic equivalent) that circulates in the blood and recognizes foreign substances like bacteria and viruses
Bacteriophages and phage-derived enzymes	Substances that directly cause pathogen lysis that are phage-derived recombinant enzymes or phages (including those engineered as nano-delivery vehicles)
Microbiome-modulating agents	Approaches that seek to modify the microbiome to eliminate or prevent carriage of resistant or pathogenic bacteria manipulating the metabolism of microbiota
Immunomodulating agents	Compounds that augment, stimulate, or suppress host immune responses that modify the course of infection
Miscellaneous agents	Group of strategies that seek to (i) inhibit the production or the activity of virulence factors such as toxins, (ii) impede bacterial adhesion to host cells and biofilm formation, (iii) interrupt or inhibit bacterial communication, and (iv) inhibit resistance mechanisms

**FIG 2:**
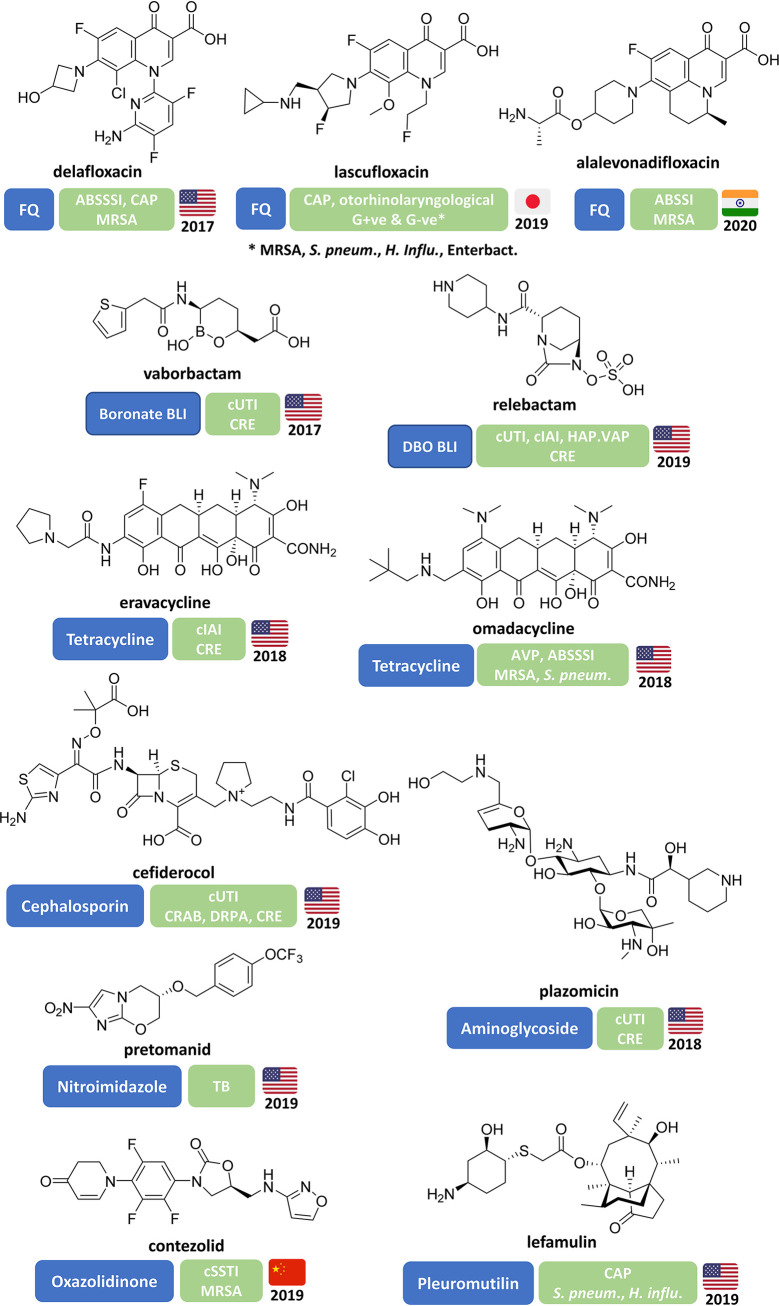
Structures of antibacterial drugs approved worldwide since 2017 and their approved indications and targeted priority pathogens with country and year of first approval.

**FIG 3:**
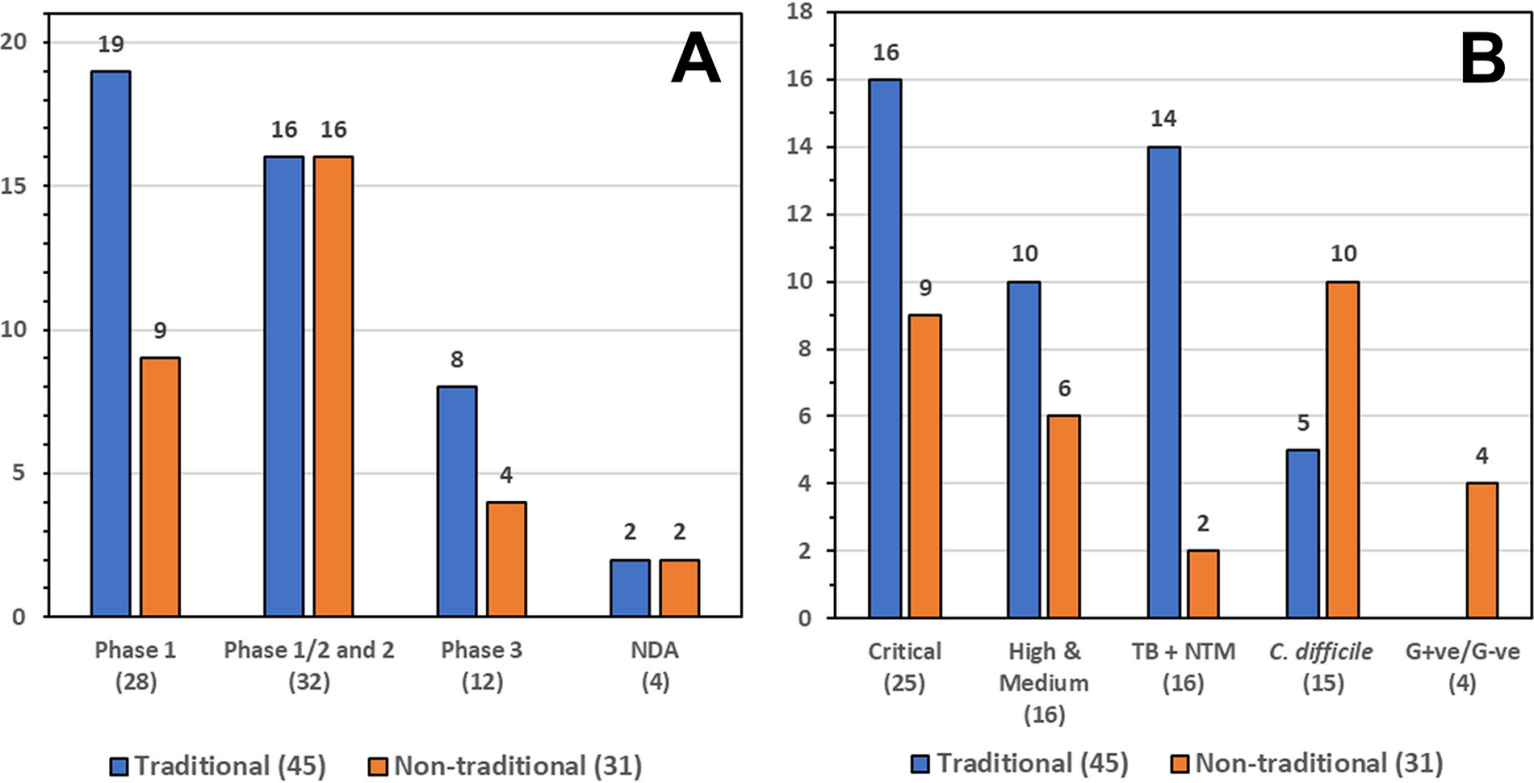
Number of traditional and nontraditional antibacterials by (A) development phase and (B) development against WHO priority pathogens, TB and NTM, C. difficile, and G+ve/G−ve.

**FIG 4:**
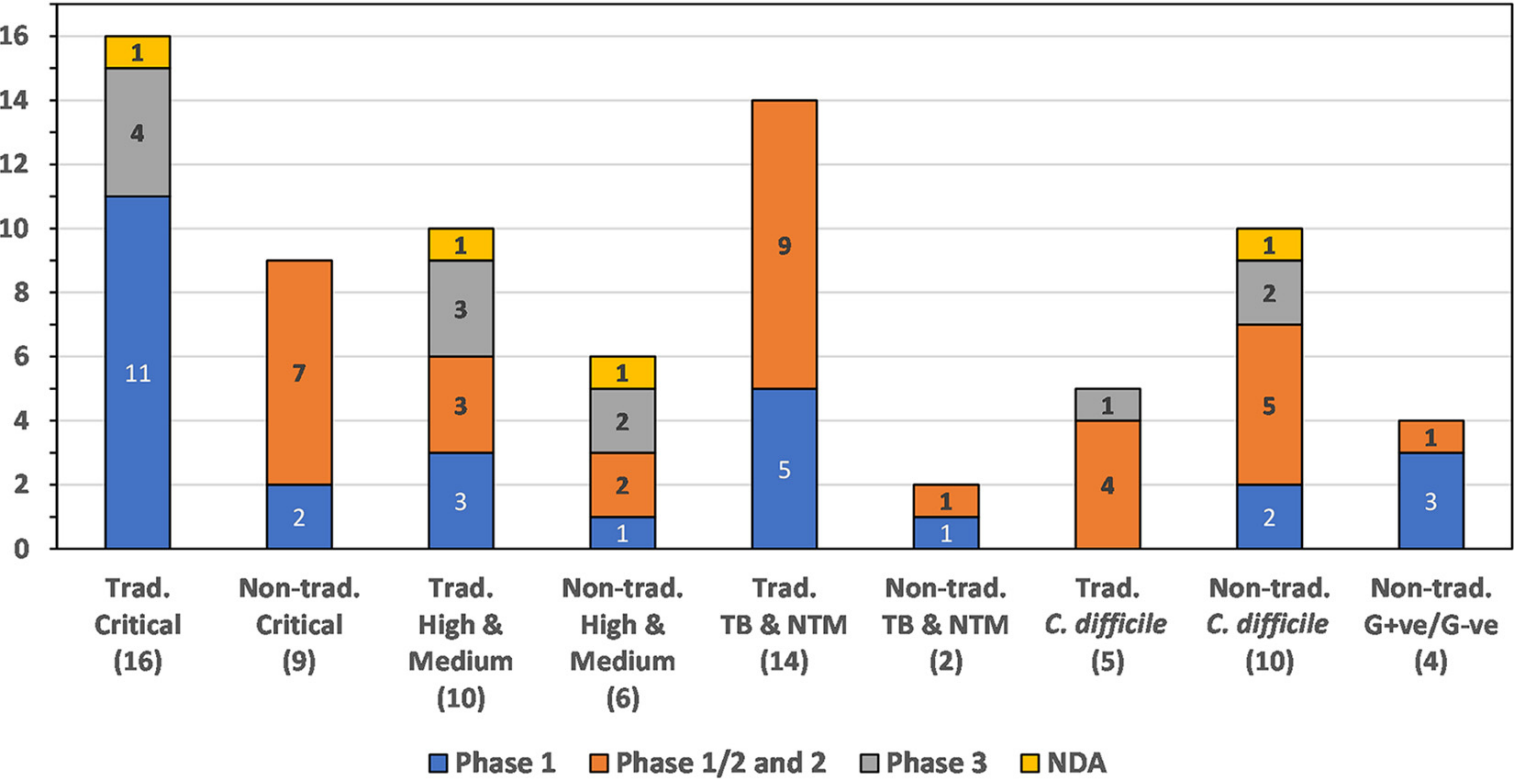
Traditional and nontraditional antibacterials categorized by development phase and activity against WHO critical pathogens, WHO high- and medium-priority pathogens TB and NTM, C. difficile, and nontraditional nonspecific G+ve/G−ve activity.

**FIG 5:**
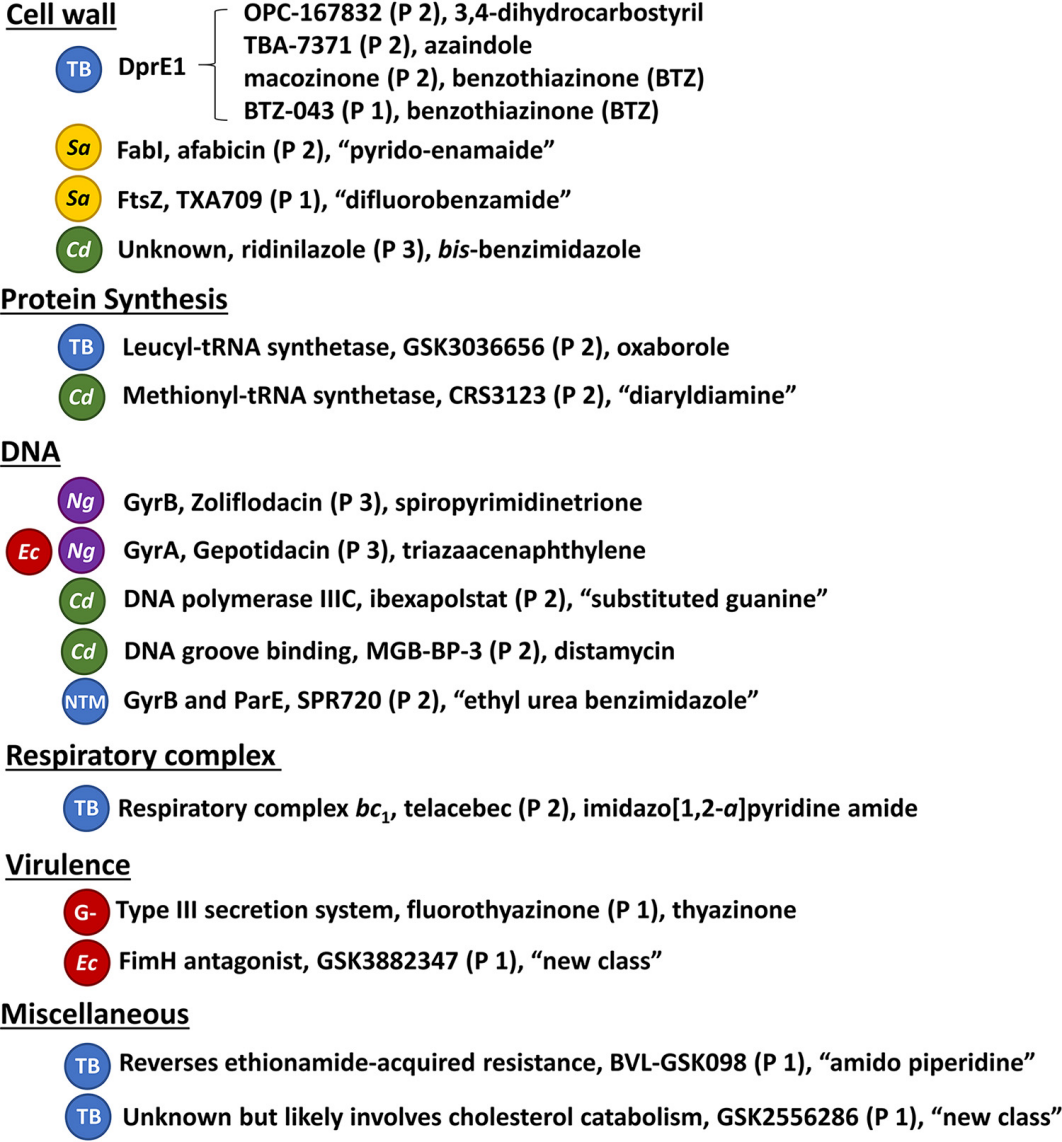
Antibacterials with new pharmacophores not previously found in human antibacterial drugs by target class, target, antibacterial name (current development phase), and antibacterial class. Abbreviations: TB, tuberculosis; Sa, S. aureus; Cd, C. difficile, Ec, E. coli; Ng, N. gonorrhoeae; NTM, nontuberculosis mycobacteria; G−, Gram-negative bacteria.

In this review, we discuss traditional and nontraditional antibacterial agents that were being evaluated in clinical trials on 30 June 2021 for the treatment of infections caused by the WHO priority pathogens Mycobacterium tuberculosis (38) and nontuberculosis mycobacteria (NTM) (39) and C. difficile, which is not a WHO priority pathogen but is considered by the CDC to be an urgent threat (11). A brief overview of the drug development process and drug regulatory agencies are provided in the supplemental information. Data for this review were based on the WHO’s antibacterial agents in clinical development reports published in 2021 (10), 2018 (8), 2019 (9), and 2017 (7), as well as WHO’s preclinical pipeline analyses (10, 40). First, we examined antibacterial drugs that had been approved anywhere in the world between 1 July 2017 and 30 June 2021 (Table 2, Fig. 2). Next, we analyzed the traditional and nontraditional antibacterial agents being evaluated in phase 1 to 3 clinical trials or those having a new drug application (NDA)/market authorization application (MAA) submitted to a regulatory body with a cutoff date of 30 June 2021 that had not previously been granted market authorization for human use anywhere in the world (Tables 3 to 8). The traditional and nontraditional antibacterial drug candidates were then analyzed by development phase (Fig. 3), target organism type (Fig. 4), and new pharmacophore types (Fig. 5).

**TABLE 2:** Antibacterial drugs that gained market authorization between July 2017 and June 2021^*a*^

Name (trade name)	Market authorization holder(s)	Agency/agencies granting approval (date)	Antibacterial class	Route of administration	Indication(s)	WHO EML & AWaRe	Expected activity against priority pathogens^*b*^				Innovation^*c*^			
							CRAB	CRPA	CRE	OPP	NCR	CC	T	MoA
Delafloxacin (Baxdela)	Melinta (Menarini, EU)	FDA (6/2017 ABSSSI, 10/2019 CAP), EMA (12/2019 ABSSSI)	Fluoroquinolone	i.v. & oral	ABSSSI, CAP	AWaRe: Watch	○	○	○	●	—	—	—	—
Vaborbactam + meropenem (Vabomere)	Melinta (Menarini, EU)	FDA (8/2017), EMA (11/2018)	Boronate BLI + β-lactam (carbapenem)	i.v.	cUTI	WHO EML & AWaRe: Reserve	○	○	●^*d*^	NA	?^*e*^	✓	—	—
Plazomicin (Zemdri)	Achaogen (Cipla USA / QiLu Antibiotics, China)	FDA (8/2018)	Aminoglycoside	i.v.	cUTI	WHO EML & AWaRe: Reserve	○	○	●	NA	—	—	—	—
Eravacycline (Xerava)	Tetraphase (La Jolla, Everest Medicines)	FDA (8/2018), EMA (9/2018)	Tetracycline	i.v.	cIAI	AWaRe: Reserve	?	○	●	NA	—	—	—	—
Omadacycline (Nuzyra)	Paratek	FDA (10/2018)	Tetracycline	i.v. & oral	CAP (iv), ABSSSI (iv, oral)	AWaRe: Reserve	○	○	○	●	—	—	—	—
Relebactam + imipenem/cilastatin (Recarbrio)	MSD	FDA (7/2019 cUTI/cIAI, 7/2020 HAP/VAP), EMA (2/2020 Gram -ve)	DBO-BLI + β-lactam (carbapenem)/ degradation inhibitor	i.v.	cUTI, cIAI, HAP/VAP	AWaRe: Reserve	○	?	●^*d*^	NA	—	—	—	—
Lefamulin (Xenleta)	Nabriva (Sunovion Pharmaceuticals Canada)	FDA (8/2019), EMA (7/2020)	Pleuromutilin	i.v. & oral	CAP	AWaRe: Reserve	NA	NA	NA	●	?	✓^*f*^	—	—
Pretomanid (Dovprela)	Viatris (TB Alliance)^*g*^	FDA (8/2019), EMA (8/2020), CDSCO (7/2020)	Nitroimidazole	Oral	XDR-TB		NA	NA	NA	●^*h*^	—	—	—	—
Lascufloxacin (Lasvic)	Kyorin Pharmaceutical	PDMA (8/2019)	Fluoroquinolone	i.v. & oral	CAP, otorhinolaryngological	AWaRe: Watch	○	○	○	●	—	—	—	—
Cefiderocol (Fetroja)	Shionogi	FDA (11/2019, cUTI; 9/21 HAP/VAP), EMA (4/2020)	Siderophore β-lactam (cephalosporin)	i.v.	FDA: cUTI, HAP/VAP, EMA: aerobic G-ve	WHO EML & AwaRe: Reserve	●	●	●	NA	?	—	—	—
Levonadifloxacin (Emrok), Alalevonadifloxacin (Emrok-O)	Wockhardt	CDSCO (1/2020)	Fluoroquinolone	i.v. & oral	ABSSSI	AwaRe: Watch	○	○	○	●	—	—	—	—
Contezolid (Youxitai), Contezolid acefosamil	MicuRx	NMPA (6/2021)	Oxazolidinone	i.v. & oral	cSSTI		NA	NA	NA	●	—	—	—	—

a Abbreviations: ABSSSI, acute bacterial skin and skin structure infections; AwaRe, Access Watch Reserve; CAP, community-acquired pneumonia; CC, new chemical class; cIAI, complicated interabdominal infection; CRAB, carbapenem-resistant Acinetobacter baumannii; CRE, carbapenem-resistant *Enterobacterales*; CRPA, carbapenem-resistant P. aeruginosa; CDSCO, Central Drugs Standard Control Organization of the Government of India; cSSTI, complicated skin and soft tissue infections; cUTI, complicated urinary tract infection; EMA, European Medicines Agency; EML, essential medicines list; FDA, Food and Drug Administration (USA); HAP, hospital-acquired pneumonia; i.v., intravenous; KPC, K. pneumoniae carbapenemase; MBL, metallo-β-lactamase; OPP, other priority pathogens; MoA, new mode of action; NCR, no cross-resistance to other antibiotic classes; NMPA, China National Medical Products Administration; PDMA, Pharmaceuticals and Medical Devices Agency (Japan); T, new target; VAP, ventilator-acquired pneumonia; XDR, extensively drug-resistant.

b Pathogen activity: ●, active; ?, possibly active; ○, not or insufficiently active; NA, activity not assessed, as the antibiotic is focused and developed for only either Gram-positive cocci or Gram-negative rods. Agents not active against critical-priority pathogens were assessed for activity against other priority pathogens (OPP), which includes the high and medium WHO priority pathogens.

c Innovation assessment: ✓, criterion fulfilled; ?, inconclusive data; —, criterion not fulfilled.

d Active against KPC- but not MBL-producing *Enterobacteriaceae*.

e Cross-resistance can be obtained when the levels of the porin OmpK36 are varied.

f First systemic formulation of this class, which was previously used in animals and topically in humans.

g The approvals were obtained by the TB Alliance and then transferred to Viatris.

h Approved for the treatment of XDR-TB or treatment-intolerant/nonresponsive MDR-TB, in combination with bedaquiline and linezolid.

**TABLE 3:** Traditional antibacterial agents and combinations in NDA and phase 3 clinical development against WHO priority pathogens

Name (synonym)	Phase	Antibacterial class	Route of administration	Developer	Expected activity against priority pathogens^*a*^				Innovation^*b*^			
					CRAB	CRPA	CRE	OPP	NCR	CC	T	MoA
Solithromycin (T-4288)	NDA^*c*^	Macrolide	i.v. & oral	iFUJIFILM Toyama Chemical	NA	NA	NA	●^*d*^	—	—	—	—
Sulopenem, Sulopenem etzadroxil/probenecid	NDA^*e*^	β-Lactam (penem)	i.v. & oral	Iterum	○	○	○^*f*^	NA	—	—	—	—
Durlobactam (ETX-2514) + sulbactam	3	DBO-BLI/PBP2 binder + β-lactam-BLI/PBP1,3 binder	i.v.	Entasis	●	○	○	NA	—	—	—	—
Taniborbactam (VNRX-5133) + cefepime	3	Boronate BLI + β-lactam (cephalosporin)	i.v.	VenatoRx/GARDP	○	●	●	NA	?	✓	—	—
Enmetazobactam (AAI-101) + cefepime	3	BLI + β-lactam (cephalosporin)	i.v.	Allecra	○	○	○^*g*^	NA	—	—	—	—
Zoliflodacin	3	Spiropyrimidenetrione (topoisomerase inhibitor)	Oral	Entasis/GARDP	NA	NA	NA	●^*d*^	✓	✓	—	✓
Gepotidacin	3	Triazaacenaphthylene (topoisomerase inhibitor)	i.v. & oral	GSK	NA	NA	NA	●^*d*^	?	✓/?^*h*^	—	✓
Nafithromycin (WCK-4873)	3	Macrolide	Oral	Wockhardt	NA	NA	NA	●^*d*^	—	—	—	—
Benapenem	2/3	β-Lactam (carbapenem)	i.v.	Sichuan Pharmaceutical	○	○	○	NA	—	—	—	—

a Pathogen activity: ●, active; ?, possibly active; ○, not or insufficiently active; NA, activity not assessed, as the antibiotic is focused and developed for only either Gram-positive cocci or Gram-negative rods. Agents not active against critical-priority pathogens were assessed for activity against OPP, which includes the high and medium WHO priority pathogens.

b Innovation assessment: ✓, criterion fulfilled; ?, inconclusive data; —, criterion not fulfilled. CC, chemical class; MOA, new mode of action; NCR, no cross-resistance; T, new target.

c Solithromycin NDA for otorhinolaryngological infections submitted in Japan in April 2019.

d OPP target pathogens: solithromycin, S. pneumoniae; nafithromycin, S. aureus and S. pneumoniae; gepotidacin, N. gonorrhoeae and E. coli; zoliflodacin, N. gonorrhoeae.

e Sulopenem etzadroxil NDA submitted in USA for uncomplicated UTI (uUTI) in November 2020.

f Active against ESBL-producing cephalosporin-resistant but not carbapenem-resistant *Enterobacterales.*

g Active against ESBL-producing cephalosporin-resistant and some KPC-producing CRE.

h Gepotidacin is being tested in two distinct phase 3 programs: gonorrhea (NCR ✓) and uUTI (NCR ?).

**TABLE 4:** Traditional antibacterial agents and combinations in phase 1 and 2 clinical development against WHO priority pathogens

Name (synonym)	Phase	Antibacterial class	Route of administration	Developer	Expected activity against priority pathogens^*a*^				Innovation^*b*^			
					CRAB	CRPA	CRE	OPP	NCR	CC	T	MoA
Afabicin (Debio-1450)	2	Pyrido-enamide (FabI inhibitor)	i.v. & oral	Debiopharm	NA	NA	NA	●^*c*^	✓	✓	✓	✓
TNP-2092	2	Rifamycin-quinolizinone hybrid	i.v. & oral	TenNor Therapeutics	NA	NA	NA	●^*c*^	—	—	—	—
TNP-2198	1b/2a	Rifamycin-nitroimidazole conjugate	Oral	TenNor Therapeutics	NA	NA	NA	●^*c*^	—	—	—	—
Zidebactam + cefepime	1^*d*^	DBO-BLI/PBP2 binder ^*e*^ + cephalosporin	i.v.	Wockhardt	●	●	●	NA	—	—	—	—
Nacubactam (OP0595) + meropenem	1	DBO-BLI/PBP2 binder ^*e*^ + β-lactam (carbapenem)	i.v.	Meiji Seika	○	○^*f*^	●	NA	—	—	—	—
ETX0282 + cefpodoxime	1	DBO-BLI/PBP2 binder ^*e*^ + β-lactam (cephalosporin)	Oral	Entasis Therapeutics	○	○	●	NA	—	—	—	—
XNW-4107+ imipenem + cilastatin	1	BLI + β-lactam (carbapenem) / degradation inhibitor	i.v.	Sinovent	?	?	?	?	?	?	?	?
VNRX-7145 + ceftibuten	1	Boronate BLI + β-lactam (cephalosporin)	Oral	VenatoRx Pharmaceuticals	○	○	●	NA	?	✓	—	—
SPR-206	1	Polymyxin	i.v.	Spero Therapeutics	●	●	●	NA	—	—	—	—
MRX-8	1	Polymyxin	i.v.	MicuRx	●	●	●	NA	—	—	—	—
QPX-9003	1	Polymyxin	i.v.	Qpex Biopharma	?	?	?	?	?	?	?	?
KBP-7072	1	Tetracycline	Oral	KBP BioSciences	●	○	○	●^*c*^	—	—	—	—
EBL-1003 (apramycin)	1^*f*^	Aminoglycoside	i.v.	Juvabis	●	○	●	NA	—	—	—	—
TXA-709	1	“Difluorobenzamide” (FtsZ inhibitor)	i.v. & oral	TAXIS Pharmaceutical	○	○	○	●^*c*^	✓	✓	✓	✓
ARX-1796 (oral avibactam prodrug)	1	DBO-BLI + β-lactam (undisclosed)	Oral	Arixa/Pfizer^*g*^	○	○	●^*h*^	NA	—	—	—	—
PLG0206 (WLBU2)	1	Cationic peptide	i.v.^*h*^	Peptilogics	?^*i*^	?^*i*^	?^*i*^	●^*c*^^,^^*j*^	?	✓	?	?
QPX7728^*k*^ + QPX2014 / QPX7728 + QPX2015	1	Boronate-BLI + β-lactam (undisclosed) / boronate-BLI + β-lactam (undisclosed)	i.v. / i.v. & oral	Qpex Biopharma	●	●	●	NA	?	—	—	—

a Pathogen activity: ●, active; ?, possibly active; ○, not or insufficiently active; NA, activity not assessed, as the antibiotic is focused and developed for only either Gram-positive cocci or Gram-negative rods. Agents not active against critical-priority pathogens were assessed for activity against OPP, which includes the high and medium WHO priority pathogens.

b Innovation assessment: ✓, criterion fulfilled; ?, inconclusive data; —, criterion not fulfilled. CC, chemical class; MOA, new mode of action; NCR, no cross-resistance; T, new target.

c OPP target pathogens: TNP-2198, H. pylori; afabicin, TNP-2092, KBP-7072, TXA-109, and PLG0206, S. aureus.

d A phase 3 trial for zidebactam + cefepime was registered in July 2021 for cUTI or acute pyelonephritis (NCT04979806).

e The DBO-BLIs zidebactam, nacubactam, and ETX0282 also have some antibacterial activity and have been classified as β-lactam enhancers (BLE) (97–99).

f Previously used as an antibacterial treatment in animals.

g Activity against AmpC-producing and KPC-producing CRPA. Active against KPC- but not MBL-producing *Enterobacteriaceae*.

h The original developer, Arixa Pharmaceuticals, was acquired by Pfizer in October 2020.

i PLG0206 was evaluated in phase 1 using i.v. administration, but development is currently focused on use as an irrigation solution for prosthetic joint infections.

j Peptilogics recently reported that coagulase-negative staphylococci, E. coli, Enterobacter cloacae, Citrobacter freundii, P. aeruginosa, and A. baumannii (100).

k QPX7728 is being evaluated with two separate β-lactams, QPX-2014 and QPX2015.

**TABLE 5:** Traditional antibacterial agents in clinical development for the treatment of TB and nontuberculous mycobacteria (NTM)

Name (synonym)	Phase	Antibiotic class	Route of administration	Developer	Innovation^*a*^			
					NCR	CC	T	MoA
GSK-3036656 (GSK070)	2	Oxaborole (Leu-Rs inhibitor)	Oral	GSK	✓	✓	✓	✓
Delpazolid (LCB01-0371)	2b	Oxazolidinone	Oral	LegoChem Biosciences/HaiHe Biopharma	—	—	—	—
Sutezolid	2	Oxazolidinone	Oral	TB Alliance/Sequella	—	—	—	—
Telacebec (Q-203)	2	Imidazopyridine amide	Oral	Qurient	✓	✓	✓	✓
TBA-7371	2	Azaindole (DprE1 inhibitor)	Oral	TB Alliance/Bill & Melinda Gates Foundation/Foundation for Neglected Disease Research	✓	✓	✓	✓
SPR720	2a^*b*^	Benzimidazole ethyl urea (GyrB inhibitor^*c*^)	Oral	Spero/Bill & Melinda Gates Foundation	—	✓	—	—
TBI-166 (pyrifazimine)^*d*^	2	Riminophenazine (clofazimine-analogue)	Oral	Institute of Materia Medica/TB Alliance/Chinese Academy of Medical Sciences/Peking Union Medical College	—	—	—	—
OPC-167832	1/2	3,4-Dihydrocarbostyril (DprE1 inhibitor)	Oral	Otsuka	✓	✓	✓	✓
BTZ-043	1/2	Benzothiazinone (DprE1 inhibitor)	Oral	University of Munich/Hans Knöll Institute, Jena/German Center for Infection Research	✓	✓	✓	✓
Macozinone (PBTZ-169)	1	Benzothiazinone (DprE1 inhibitor)	Oral	Innovative Medicines for Tuberculosis Foundation/Nearmedic Plus	✓	✓	✓	✓
TBI-223	1	Oxazolidinone	Oral	TB Alliance/Institute of Materia Medica	—	—	—	—
TBAJ-876	1	Diarylquinoline	Oral	TB Alliance	—	—	—	—
TBAJ-587	1	Diarylquinoline	Oral	TB Alliance	—	—	—	—
GSK 2556286 (GSK-286)	1	Undisclosed	Oral	GSK/TB Drug Accelerator/Bill & Melinda Gates Foundation	?	✓	✓	?

a Innovation assessment: ✓, criterion fulfilled; ?, inconclusive data; —, criterion not fulfilled. CC, chemical class; MOA, new mode of action; NCR, no cross-resistance; T, new target.

b This phase 2a trial (NCT04553406) was on FDA clinical hold, but this was lifted in January 2022.

c This is not considered to be a new mode of action, as the GyrB/ParE inhibitor novobiocin was once marketed but is no longer in clinical use.

d The lead drug clofazimine is approved to treat leprosy and has been used off-label for TB treatment.

**TABLE 6:** Traditional antibacterial agents in clinical development for the treatment of C. difficile infections

Name (synonym)	Phase	Antibiotic class	Route of administration	Developer	Innovation^*a*^			
					NCR	CC	T	MoA
Ridinilazole	3	*Bis*-benzimidazole	Oral	Summit Therapeutics	✓	✓	✓	✓
DNV-3837 (MCB-3837)	2	Oxazolidinone-quinolone hybrid	i.v.	Deinove	?	—	—	—
MGB-BP-3	2	Distamycin (DNA minor groove binder)	Oral	MGB Biopharma	?	✓	✓	✓
Ibezapolstat (ACX-362E)	2	“Substituted guanine” (DNA polymerase IIIC inhibitor)	Oral	Acurx Pharmaceuticals	?	✓	✓	✓
CRS3123	2	“Diaryldiamine” (methionyl-tRNA synthetase inhibitor; MetRS)	Oral	Crestone/NIAID	✓	✓	✓	✓

a Innovation assessment: ✓, criterion fulfilled; ?, inconclusive data; —, criterion not fulfilled. CC, chemical class; MOA, new mode of action; NCR, no cross-resistance; T, new target. These agents are being developed for C. difficile infections, and their activity against WHO priority pathogens was not assessed.

**TABLE 7:**
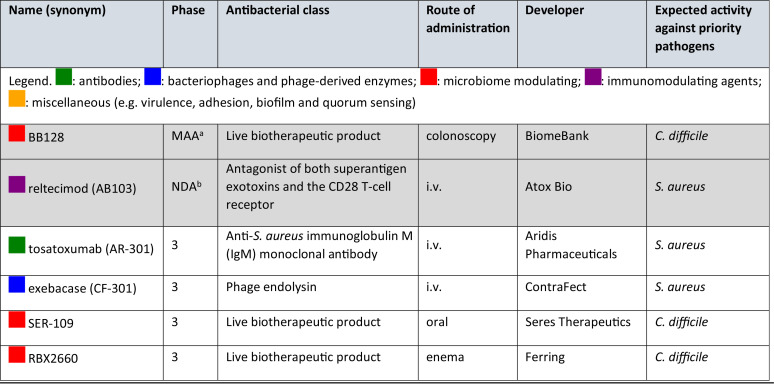
Nontraditional antibacterial agents in phase 3 clinical development

a Submitted to the Australian Therapeutic Goods Association (TGA) as a potential treatment for recurrent C. difficile infections in June 2021.

b Submitted to the U.S. FDA as a potential treatment for necrotizing soft tissue infections (NSTI) in December 2020.

**TABLE 8:**
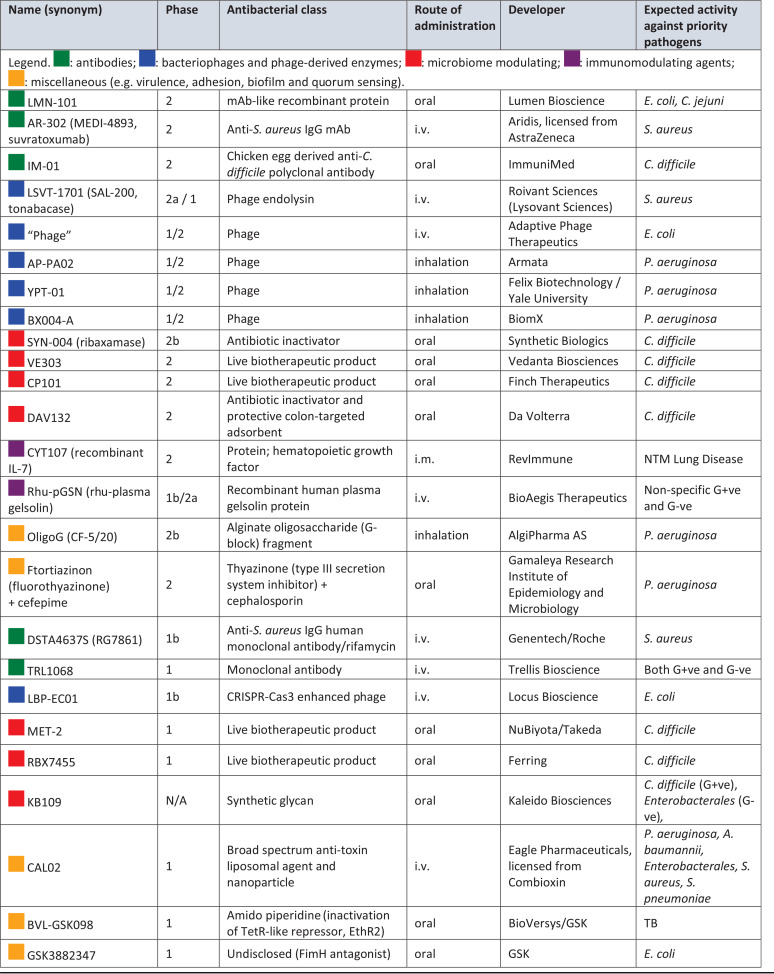
Nontraditional antibacterial agents in phase 1 and 2 clinical development for WHO priority pathogens, mycobacteria, and C. difficile

## METHODOLOGY

### Scope and inclusion/exclusion criteria.

This review details the antibacterial drugs that have been approved for the treatment of WHO priority pathogens anywhere in the world between 1 July 2017 and 30 June 2021. Also included in this analysis are traditional and nontraditional antibacterial agents administered by intravenous (i.v.), intramuscular (i.m.), oral, inhalation, enema, and colonoscopy administration routes that are currently being evaluated in phase 1 to 3 clinical trials or have NDA/MAA applications under consideration that have not previously been granted market authorization for human use anywhere in the world. Antibacterial agents were restricted to those being developed or that have the potential to treat bacterial infections caused by the WHO priority pathogens (Fig. 1), mycobacteria, or C. difficile and are included only if they are new chemical entities (NCEs) (traditional or nontraditional) or new biological entities (NBEs) (nontraditional) not already accorded market authorization for human use anywhere in the world. Antibacterial agents whose development programs have been terminated, are no longer listed on a company’s development pipeline, or have not had any development update for three or more years have been excluded in this analysis and are listed in the supplemental information (Table S1). This review does not include new formulations of approved antibacterial drugs, vaccines, topical decolonizing agents, nonspecific inorganic substances, and antibacterial agents developed only for topical applications such as creams, ointments, or eye drops. Fixed-dose combinations of potentiators and antibacterial agents are included if they contain an NCE or an NBE.

### Search strategy.

Data from the 2020 WHO antibacterial pipeline report (10) were used as a starting point for this updated analysis. Recent antibacterial pipeline reviews (41–43), previous WHO reports (7, 9), and The Pew Charitable Trusts’ antibiotic development pipeline reviews (6) were also consulted. Additional references were identified using searches of antibacterial compound names and their synonyms from PubMed (https://pubmed.ncbi.nlm.nih.gov/), Google Scholar (https://scholar.google.com.au/), and conference abstracts and posters. The U.S. NIH (https://clinicaltrials.gov/) and WHO International Clinical Trials Registry Platform (ICTRP) clinical trial databases (https://www.who.int/clinical-trials-registry-platform), the commercial database AdisInsight (https://adisinsight.springer.com/), and the Access to Medicine Foundation’s Antimicrobial Resistance Benchmark 2020 Antibacterials data (44) were searched. The websites of pharmaceutical companies active in antibacterial R&D and antibacterial development funders and foundations were also searched.

## ANALYSIS AND DISCUSSION

### Antibacterial drugs approved since 2017.

Twelve new antibacterial drugs (Table 2, Fig. 2) have been approved since the WHO’s first analysis of the clinical antibacterial pipeline in 2017 (7). The most recent approval was in China in June 2021 for the oxazolidinone contezolid as a treatment for complicated skin and soft tissue infections (cSSTI) caused by Gram-positive bacteria, including methicillin-resistant Staphylococcus aureus (MRSA) and vancomycin-resistant enterococci (VRE) (45).

Only 1 of the 12 approved antibacterial drugs, the boronate β-lactamase inhibitor (BLI) vaborbactam, which is used in combination with meropenem, has a new antibacterial drug-related pharmacophore. Also, the cephalosporin cefiderocol (46, 47) is noteworthy, as it is the first marketed β-lactam (cephalosporin) that has an iron-chelating siderophore incorporated into the structure, which facilitates Gram-negative bacteria outer membrane entry. The other 10 are members of previously approved antibacterial classes: three fluoroquinolones (delafloxacin, lascufloxacin, and levonadifloxacin), two tetracyclines (eravacycline and omadacycline), one aminoglycoside (plazomicin), one pleuromutilin (lefamulin), one nitroimidazole (pretomanid), one diazabicyclooctane (DBO) BLI (relebactam), and one oxazolidinone (contezolid). Of the 12 new antibacterial drugs, 6 target carbapenem-resistant *Enterobacterales* (CRE), 5 target other WHO priority pathogens (high and medium priority), and 1 was approved to treat MDR/extensively drug-resistant tuberculosis (XDR-TB) in combination with two other drugs, bedaquiline and linezolid. However, it is noted that lefamulin (48) is the first systemically administered pleuromutilin approved for human use (retapamulin was approved for human topical use and valnemulin and tiamulin for veterinary medicine).

### Traditional antibacterial agents in phase 3 clinical trials or with NDAs submitted against WHO priority pathogens.

We identified two compounds, solithromycin and sulopenem, that have had NDAs submitted to the Japanese PMDA and U.S. FDA, respectively. Although after this review’s cutoff date, in late July 2021, the FDA indicated that sulopenem will require further clinical trials to be undertaken (49). Three BLI/β-lactam combinations (durlobactam/sulbactam, taniborbactam/cefepime, and enmetazobactam/cefepime) and two compounds being developed to treat Neisseria gonorrhoeae infections (zoliflodacin and gepotidacin) are currently in phase 3 trials (Table 3). Gepotidacin is also being evaluated in a phase 3 trial to treat urinary tract infections (UTIs). Updating the 2020 WHO report (10), nafithromycin is now being evaluated in a phase 3 trial (CTRI/2019/11/021964) for treatment of community-associated pneumonia (CAP) in India, and the phase 2/3 trial (NCT04505683) of benapenem in China has been completed for complicated urinary tract infections (cUTI), including acute pyelonephritis.

### Other phase 3 agents of interest: ATM-AVI and tebipenem pivoxil.

There are two antibacterial agents in phase 3 development that did not meet this review’s inclusion criteria (see “Scope and inclusion/exclusion criteria”) that are noteworthy. The first is the aztreonam (monobactam-type β-lactam) and avibactam (BLI) combination (ATM-AVI), which was not included, as both components are previously approved drugs. ATM-AVI is being studied by Pfizer in a phase 3 trial (NCT03580044) to treat serious infection due to metallo-β-lactamase (MBL)-producing Gram-negative bacteria (50, 51) with support from the Biomedical Advanced Research and Development Authority (BARDA), Innovative Medicines Initiative (IMI), and AbbVie, through their 2020 acquisition of Allergan. The second is the carbapenem prodrug tebipenem pivoxil (52, 53), which was first approved for pediatric use in Japan in 2009 but has not been used elsewhere. Spero Therapeutics recently completed a phase 3 trial (NCT03788967) for tebipenem pivoxil (SPR994) as an oral treatment for Gram-negative cUTI and acute pyelonephritis infections. Clinically, the oral administration of tebipenem pivoxil could provide an alternative to i.v. administered carbapenems.

### Traditional antibacterial agents in phase 1 and 2 clinical trials being developed against WHO priority pathogens.

We identified 2 compounds in phase 2, 1 in phase 1/2a, and 14 in phase 1 development (Table 4). Since the 1 September 2020 cutoff date of the 2020 WHO pipeline report (10), two new polymyxin derivatives, MRX-8 (54, 55) (NCT04649541) and QPX-9003 (56, 57) (NCT04808414), started phase 1 trials in November 2020 and June 2021, respectively, as potential treatments for MDR Gram-negative pathogens. XNW-4107 is a BLI being that is developed in combination with imipenem and cilastatin that started a phase 1 in June 2021 (NCT04801043). The developer, Sinovent, has indicated that the XNW-4107 combination will be developed to treat patients with CRE, CRAB, and drug-resistant Pseudomonas aeruginosa (58). The structures of MRX-8, QPX-9003, and XNW-4107 have not been publicly disclosed. Finally, the rifamycin-quinolizinone hybrid TNP-2198 has moved into a Helicobacter pylori phase 1/2a trial (59).

### Traditional antibacterial agents against M. tuberculosis and nontuberculosis mycobacteria.

There are currently 14 traditional antibacterial agents being evaluated in clinical trials against mycobacteria: 7 in phase 2, 2 in phase 1/2a, and 6 in phase 1. There also are two nontraditional antibacterial agents, CYT107 (NTM) and BVL-GSK098 (M. tuberculosis), under clinical investigation. In addition to these trials, there are also approximately 20 ongoing phase 3 trials (60) investigating new combinations and dosing regimens of previously approved TB drugs, which were not included in this review due to the selection criteria. Thirteen of the traditional antibacterial candidates target M. tuberculosis, and only one, SPR720, is in development for lung infections caused by the NTMs, Mycobacterium avium complex, and Mycobacterium abscessus (Table 5). Eight of the 14 traditional antibacterial agents belong to new classes, and 9 have new antibacterial pharmacophores (see below).

Since the 2020 WHO report (10) was released, two new compounds, TBAJ-587 (NCT04890535) and GSK 2556286 (NCT04472897), have entered phase 1 trials. TBAJ-587 is a bedaquiline analog with enhanced *in vitro* potency and a projected reduced cardiovascular liability (61, 62). GSK 2556286 (GSK-286) acts directly on M. tuberculosis, and its mode of action has not been disclosed but has been proposed to involve cholesterol catabolism (63, 64). In addition, TBI-166 (pyrifazimine) has moved to phase 2 (NCT04670120) and BTZ-043 has started a new phase 1/2 trial (NCT04044001).

### Traditional antibacterial agents being developed against C. difficile.

There are currently five traditional antibacterial agents (Table 6), one in phase 3 (ridinilazole) and four in phase 2 (DNV-3837, MGB-BP-3, ibezapolstat, and CRS3123), being developed to treat C. difficile. Antibacterial drugs used to treat C. difficile infections (CDI) are usually administered orally and absorbed poorly, as the infection is localized in the colon. This has encouraged the development of four new antibacterial classes and modes of action (ridinilazole, MGB-BP-3, ibezapolstat, and CRS3123; Fig. 5), while DNV-3837 is differentiated through its administration via i.v. infusion and is being targeted for use in patients who are unable to receive oral administration. Since the 2020 WHO report (10) was published, CRS3123 has started a phase 2 trial (NCT04781387).

### Nontraditional antibacterial agents in phase 3 clinical trials.

We found two nontraditional antibacterial agents in the NDA/MAA phase and four in phase 3 development (Table 7). Three are being developed to treat S. aureus infections: tosatoxumab is an MAb (65), exebacase is a phage-derived recombinant protein (66), and reltecimod is an immune modulator (CD28 T-lymphocyte receptor mimetic) (67). Three are being developed to treat C. difficile infections: SER-109 consists of purified *Firmicutes* spores (68), RBX2660 is a liquid suspension of screened donor fecal microbiota (69, 70), and BB128 is a lyophilized donor fecal microbiota product. SER-109 has already successfully completed one phase 3 trial (NCT03183128) (71).

Since the publication of the 2020 WHO pipeline report (10), a phase 3 trial (NCT03931941) has started to evaluate RBX2660 as a treatment of C. difficile, while BiomeBank has submitted an MAA to the Australian Therapeutic Goods Association (TGA) for BB128 as a potential treatment of recurrent C. difficile and ulcerative colitis (72). BiomeBank already has provisional approval for its use in Australia as a class 2 biologic. In addition, Atox Bio applied for an NDA in December 2020 for reltecimod as a potential supportive treatment for necrotizing soft tissue infections (NSTI) (73).

### Nontraditional antibacterial agents in phases 1 and 2.

There are 10 nontraditional antibacterial agents in phase 2, 6 in phase 1/2a, 8 in phase 1 clinical trials, and 1 not disclosed by clinical phase (Table 8). Combined with the 4 in phase 3 and 2 at the NDA/MAA stage, there are 31 nontraditional agents overall in clinical development.

There are seven nontraditional antibacterial agents not detailed in the 2020 WHO report (10): CYT107, TRL1068, BVL-GSK098, and four bacteriophage products. CYT107 is a glycosylated recombinant human interleukin (IL-7) that is being tested in a phase 2 trial (NCT04154826) to evaluate its immunotherapeutic response in patients with NTM lung disease. CYT107 has been evaluated in other clinical trials, including a phase 2b trial (NCT02640807) that reported a 3- to 4-fold increase in the absolute lymphocyte count and in circulating CD4^+^ and CD8^+^ T cells with CYT107 in sepsis patients (predominantly secondary to pneumonia and abdominal infections) (74). TRL1068 is an MAb that binds to a DNABII epitope conserved across both Gram-positive and Gram-negative bacteria, which leads to bacterial biofilm disintegration, and is being evaluated in a phase 1 trial (NCT04763759) for prosthetic joint infections (75, 76). BVL-GSK098 recently entered phase 1 (NCT04654143) and works through inactivation of a TetR-like repressor, EthR2, thereby enhancing ethionamide activation (77). BVL-GSK098 is intended to be used clinically in combination with ethionamide or prothionamide (78). Adaptive Phage Therapeutics are undertaking a phase 1/2 trial (APT.UTI.001, NCT04287478) to evaluate its PhageBank therapy in patients with UTI. There are also three other phage products, AP-PA02 (NCT04596319), YPT-01 (NCT04684641), and BX004-A (NCT05010577), being evaluated in phase 1/2 trials with cystic fibrosis patients with P. aeruginosa infections. For the phage-derived endolysin tonabacase (N-Rephasin SAL200), a new phase 1 trial has been initiated and it has been renamed LSVT-1701 (79, 80).

### Antibacterial candidates in clinical trials with new pharmacophores.

Although there have been significant efforts to identify antibacterial agents with new modes of action, most marketed antibacterial drugs still fall into four overarching mechanistic classes: inhibition of cell envelope biogenesis, DNA homeostasis, RNA homeostasis, and protein synthesis (81). A pharmacophore describes the part of a molecular structure that is responsible for a particular biological or pharmacological activity and is a key component, along with antibacterial activity differences, to decide whether an antibacterial agent belongs to a new class or subclass of antibiotics. It is possible to have antibacterial drugs and clinical candidates with the same mode of action but with different pharmacophores, which can have significant effects on biological activity, metabolism, and pharmacokinetics. For example, there are four compounds in clinical development that inhibit the *M. tuberculosis* cell wall synthesis enzyme decaprenylphosphoryl-β-d-ribose 2′-epimerase (DprE1) that have three distinct pharmacophores: benzothiazinone (BTZ), azaindole, and 3,4-dicarbostyril (Fig. 5, Fig. S2).

There are 19 antibacterial agents with 18 new pharmacophores (macozinone and BTZ-043 are both BTZs) with seven inhibiting cell envelope synthesis, two acting at the protein synthesis level and five affecting DNA synthesis (Fig. 5, structures in supplemental information Fig. S1 to S3). Telacebec inhibits the mycobacterial respiratory system (82, 83), which was first targeted by bedaquiline, via inhibition of the respiratory complex *bc*_1_ (84). Half of the 18 new pharmacophores target mycobacteria, and 4 target C. difficile.

Four of 19 antibacterial agents have new overarching modes of actions not previously exploited by marketed antibacterial drugs. Two target virulence: fluorothyazinon (phase 2, NCT03638830) and GSK 3882347 (phase 1, NCT04488770). Ftortiazinon inhibits the Gram-negative type III secretion system (85) and is being evaluated in a trial in combination with cefepime, and GSK 3882347 functions as an antagonist of the Gram-negative type 1 pilus adhesin (FimH) (86). BVL-GSK098 (phase 1, NCT04654143) (77) directly inhibits ethionamide-acquired resistance, while GSK 2556286 (phase 1 trial, NCT04472897) is proposed to involve *M. tuberculosis* cholesterol catabolism (63, 64).

### Antibacterial agents that have halted or stopped clinical development.

Drug development is inherently risky, and it is not uncommon for clinical development programs to be terminated or halted. The most common reasons for stopping development include lack of clinical efficacy, off-target toxicity, and commercial considerations (87). While antibacterial agents can drop out of the pipeline due to efficacy and resistance issues, it is more common to be due to toxicity and commercial concerns (16, 41, 88). A list of antibacterial compounds and nontraditional moieties whose development has been terminated or halted are listed in the supplemental information (Table S1).

### Current pipeline analysis.

There are 76 antibacterial agents in clinical development using this review’s inclusion criteria, which are divided into 45 traditional and 31 nontraditional antibacterial agents (Fig. 3). Seventy-nine percent (60/76) of the antibacterial drug candidates are in phase 1 (28) and phase 2 (32), but as expected, this number falls away for late-stage development agents (12 in phase 3 and 4 NDA/MAA). This relatively low number of candidates in the later stages of drug development generally reflects the usual level of attrition in the pipeline, which is caused by several factors, including lack of efficacy, unacceptable toxicity, and market factors (87, 88). The number of early development candidates is encouraging and reinforces research efforts and recent funding that have been invested into discovery and preclinical development. For example, CARB-X has funded 92 early-stage R&D drug and diagnostics projects since its inception 5 years ago (89), while GARDP (90) has signed license and codevelopment agreements with the companies that are developing two innovative products, zoliflodacin (target: gonorrhea) and cefepime-taniborbactam (target: cUTI). In addition, the AMR Action Fund plans to help support late-stage development of 3 to 4 new antibacterial candidates by 2030, which could help increase the number of new approvals (17, 91).

The fact that there are 76 antibacterial candidates currently being evaluated in clinical trials is promising, but it needs to be asked whether these agents will address future clinical needs. To evaluate this, pipeline agents are analyzed here for activity versus each of the major pathogen categories.

### The potential impact of nontraditional antibacterial agents.

Nontraditional antibacterial agents have the potential to improve the clinical outcomes using alternative mechanisms to traditional antibacterial drugs. Although the number of nontraditional antibacterial agents entering clinical trials continues to increase, only one has been approved that successfully completed phase 3 trials: bezlotoxumab (34), which is a C. difficile toxin B-binding MAb. One of the main issues facing nontraditional agent developers has been clinical trial design (32, 33), except for adjunctive agents that are being developed in combination with standard of care drugs.

### WHO priority pathogens.

A total of 26/76 (34%) and 16/76 (21%) antibacterial agents are being developed to target the critical- and high/medium-priority WHO priority pathogens, respectively. This represents 55% of the total pipeline, and it is encouraging to observe product development being directed against the key pathogens. For the traditional antibacterial agents, only two compounds, gepotidacin and zoliflodacin, which are both new chemical classes, target priority pathogens (E. coli critical and N. gonorrhoeae high). β-Lactams, with and without BLI inhibitors, account for a majority of the other antibacterial agents in development against critical-priority pathogens.

Although there are six nontraditional agents in late-stage clinical development (Table 7), only three of these target the high-priority pathogen S. aureus; although there is need for innovative drugs to treat S. aureus infection, there are already several treatment options currently available to clinicians. There are nine agents in phase 1 and 2 trials being developed against critical WHO priority pathogens: four bacteriophages, one CRISP-Cas3 enhanced phage (LBP-EC01), two antivirulence (ftortiazinon and GSK-3882347), MAb-like recombinant protein (LMN-01), and an alginate oligosaccharide fragment (OligoG). Antibacterial developers and funders need to continue to develop pathways that allow the most promising of these antibacterial agents to rapidly progress through to late-stage clinical trials and beyond.

### TB and NTM.

There are 16/76 (21%) candidates being developed to treat mycobacterial infections (14 TB and 2 NTM), which includes nine small molecules with new pharmacophores (Fig. 5, Fig. S2). Despite the considerable challenges associated with TB drug development (92), progress has been accelerated from sustained funding and guidance by organizations such as the TB Alliance and the Gates Foundation (93). The next challenge will be to move the most promising candidates through the pipeline and select and clinically evaluate the optimal drug regimens.

### C. difficile.

There are also 15/76 (20%) agents in development to treat C. difficile infections, with 10 of these being nontraditional and 5 traditional antibacterial agents. Four of the five traditional antibacterial agents would be new classes if approved (Fig. S3). Of the 10 nontraditional agents, 7 are biotherapeutic products, 1 is an MAb (IM-01), and 2 are antibiotic inactivators (ribaxamase and DAV132). There are already several C. difficile drugs on the market, and it will be interesting to monitor the impact of any new approvals of small molecular antibacterial drugs and nontraditional biotherapeutic products and the effect that these will have on clinical practice and the market.

### Broad-spectrum agents active against Gram-negative and Gram-positive bacteria.

Four of the 76 (5%) are nontraditional antibacterial agents with broad-spectrum antibacterial effects, which was achieved through a variety of mechanisms: a recombinant gelsolin protein Rhu-pGSN boosts the immune system, the MAb TRL1068 disrupts biofilms, the synthetic glycan KB109 modulates the gut microbiome composition and metabolic output, and the liposomal agent CAL02 captures and neutralizes bacterial toxins.

## CONCLUSION

Since its release in 2017, the WHO’s priority pathogen list (Fig. 1) has become a focus for antibacterial R&D and stewardship initiatives. The WHO also started analyzing the antibacterial pipeline in 2017, and since then, only vaborbactam (boronate BLI) of the 12 approved antibacterial drugs (Table 2, Fig. 2) is not a derivative of a previously approved class. Importantly, vaborbactam is used in combination with meropenem to treat *Enterobacterales* infections, which are critical-priority pathogens. The cephalosporin derivative cefiderocol is also noteworthy, as it displays activity against all three critical-priority pathogens, CRAB, CRPA, and CRE, regardless of the carbapenemase mechanism, and is the first marketed antibacterial drug that incorporates an iron-chelating siderophore.

Renewed focus to identify new antibacterial drugs against MDR bacteria, combined with several recent financing mechanisms, has helped to increase the number of traditional and nontraditional antibacterial agents moving through the preclinical (94) and clinical development pipelines (41, 90, 95, 96). Despite a total of 76 antibacterial candidates (45 traditional and 31 nontraditional) being evaluated in clinical trials on 30 June 2021, our analysis indicated that there were still relatively few clinically differentiated antibacterial agents in late-stage clinical development, especially against critical-priority pathogens. In addition, we identified 18 new antibacterial pharmacophores, but only 2 had activity against priority pathogens with most targeted mycobacteria and C. difficile. It is important to try to keep on identifying and developing antibacterial agents with new modes of action to try to slow down antibacterial drug resistance. Furthermore, we believe that future antibacterial R&D should focus on the development of innovative and clinically differentiated candidates that have clear and feasible progression pathways right through development and onto the market. There needs to be a development focus on quality over quantity, especially with limited development resources, ever-increasing numbers of MDR infections, and potential return-on-investment issues associated with development, manufacture, regulatory compliance, and distribution costs.

Formidable challenges that we believe remain that still need further attention are as follows:

• Difficulty in discovering novel antibacterial leads with selective activity against MDR bacteria that are nontoxic and have suitable pharmacokinetic and pharmacodynamic properties, especially with new modes of action
• Current unmet medical need for new drugs to treat drug-resistant A. baumannii (e.g. CRAB) and P. aeruginosa (e.g. CRPA) infections
• Development of antibacterial agents for use in neonates and children
• Development of efficient progression pathways for nontraditional antibacterial candidates through the manufacturing, clinical trials, and approval processes
• Difficulties in optimal trial design and selection of relevant intended target population
• Sustained advocacy for strong and sustainable political support and governmental commitments to promote R&D and help developers overcome economic, scientific, and technical barriers
• Implementation of business models that improve the current market dynamics with a focus on developing and securing approval of truly innovative and clinically differentiated antibacterial treatments

## References

[1] Mattar C, Edwards S, Baraldi E, Hood J. 2020. An overview of the global antimicrobial resistance research and development hub and the current landscape. Curr Opin Microbiol 57:56–61. 10.1016/j.mib.2020.06.009. 32777653

[2] Global AMR R&D Hub. 2021. Estimating global patient needs and market potential for priority health technologies addressing antimicrobial resistance. https://globalamrhub.org/our-work/studies/market-potential-and-priority-patient-needs/. Global AMR R&D Hub, Berlin, Germany.

[3] World Health Organization. 2017. WHO publishes list of bacteria for which new antibiotics are urgently needed. https://www.who.int/news/item/27-02-2017-who-publishes-list-of-bacteria-for-which-new-antibiotics-are-urgently-needed. WHO, Geneva, Switzerland.

[4] Tacconelli E, Carrara E, Savoldi A, Harbarth S, Mendelson M, Monnet DL, Pulcini C, Kahlmeter G, Kluytmans J, Carmeli Y, Ouellette M, Outterson K, Patel J, Cavaleri M, Cox EM, Houchens CR, Grayson ML, Hansen P, Singh N, Theuretzbacher U, Magrini N, WHO Pathogens Priority List Working Group. 2018. Discovery, research, and development of new antibiotics: the WHO priority list of antibiotic-resistant bacteria and tuberculosis. Lancet Infect Dis 18:318–327. 10.1016/S1473-3099(17)30753-3. 29276051

[5] Strathdee SA, Davies SC, Marcelin JR. 2020. Confronting antimicrobial resistance beyond the COVID-19 pandemic and the 2020 US election. Lancet 396:1050–1053. 10.1016/S0140-6736(20)32063-8. 33007218 PMC7524538

[6] The Pew Charitable Trusts. 2021. Tracking the global pipeline of antibiotics in development, March 2021. https://www.pewtrusts.org/en/research-and-analysis/issue-briefs/2021/03/tracking-the-global-pipeline-of-antibiotics-in-development. The Pew Charitable Trusts, Philadelphia, PA.

[7] World Health Organization. 2017. Antibacterial agents in clinical development: an analysis of the antibacterial clinical development pipeline, including tuberculosis. World Health Organization, Geneva, Switzerland.

[8] World Health Organization. 2018. Update of antibacterial agents in clinical development 2018, Geneva, Switzerland.

[9] World Health Organization. 2019. 2019 antibacterial agents in clinical development: an analysis of the antibacterial clinical development pipeline. World Health Organization, Geneva, Switzerland.

[10] World Health Organization. 2021. 2020 antibacterial agents in clinical and preclinical development: an overview and analysis. World Health Organization, Geneva, Switzerland.

[11] Centres for Disease Control and Prevention. 2019. Biggest threats and data, 2019 antibiotic resistance threats report. https://www.cdc.gov/drugresistance/biggest-threats.html. CDC, Washington, DC.

[12] The Centers for Disease Control and Prevention. 2013. Antibiotic resistance threats in the United States, 2013. https://www.cdc.gov/drugresistance/threat-report-2013/pdf/ar-threats-2013-508.pdf. CDC, Washington, DC.

[13] WHO Country Office for India and Department of Biotechnology Government of India. 2021. Indian priority pathogen list. To guide research, discovery and development of new antibiotics in India. https://dbtindia.gov.in/sites/default/files/IPPL_final.pdf. World Health Organization, Geneva, Switzerland.

[14] Rex JH, Outterson K. 2021. Antibacterial R&D at a crossroads: we’ve pushed as hard as we can … now we need to start pulling!. Clin Infect Dis 73:e4451–e4453. 10.1093/cid/ciaa852. 32584949

[15] Rahman S, Lindahl O, Morel CM, Hollis A. 2021. Market concentration of new antibiotic sales. J Antibiot (Tokyo) 74:421–423. 10.1038/s41429-021-00414-5. 33664435

[16] Dheman N, Mahoney N, Cox EM, Farley JJ, Amini T, Lanthier ML. 2021. An analysis of antibacterial drug development trends in the United States, 1980–2019. Clin Infect Dis 73:e4444–e4450. 10.1093/cid/ciaa859. 32584952

[17] Clancy CJ, Nguyen MH. 2020. Buying time: the AMR Action Fund and the state of antibiotic development in the United States 2020. Open Forum Infect Dis 7:ofaa464. 10.1093/ofid/ofaa464. 33209952 PMC7652093

[18] Lee RA, Centor RM, Humphrey LL, Jokela JA, Andrews R, Qaseem A, Akl EA, Bledsoe TA, Forciea MA, Haeme R, Kansagara DL, Marcucci M, Miller MC, Obley AJ, Scientific Medical Policy Committee of the American College of Physicians. 2021. Appropriate use of short-course antibiotics in common infections: best practice advice from the american college of physicians. Ann Intern Med 174:822–827. 10.7326/M20-7355. 33819054

[19] Kinch MS, Patridge E, Plummer M, Hoyer D. 2014. An analysis of FDA-approved drugs for infectious disease: antibacterial agents. Drug Discov Today 19:1283–1287. 10.1016/j.drudis.2014.07.005. 25043770

[20] World Health Organization. 2020. A financial model for an impact investment fund for the development of antibacterial treatments and diagnostics. https://www.who.int/publications/i/item/a-financial-model-for-an-impact-investment-fund-for-the-development-of-antibacterial-treatments-and-diagnostics-a-user-guide. World Health Organization, Geneva, Switzerland.

[21] Gotham D, Moja L, van der Heijden M, Paulin S, Smith I, Beyer P. 2021. Reimbursement models to tackle market failures for antimicrobials: approaches taken in France, Germany, Sweden, the United Kingdom, and the United States. Health Policy 125:296–306. 10.1016/j.healthpol.2020.11.015. 33402265

[22] AMR Alliance Japan. 2021. Creating pull incentives for antimicrobials (March 24, 2021). https://hgpi.org/en/research/amr-21.html. Health and Global Policy Institute, Tokyo, Japan.

[23] International Federation of Pharmaceutical Manufacturers and Associations. 2021. Global principles on incentivizing antibiotic R&D. https://www.ifpma.org/resource-centre/global-principles-on-incentivizing-antibiotic-rd/. IFPMA, Geneva, Switzerland.

[24] Cama J, Leszczynski R, Tang PK, Khalid A, Lok V, Dowson CG, Ebata A. 2021. To push or to pull? In a post-COVID world, supporting and incentivizing antimicrobial drug development must become a governmental priority. ACS Infect Dis 7:2029–2042. 10.1021/acsinfecdis.0c00681. 33606496 PMC7931625

[25] Outterson K. 2021. Estimating the appropriate size of global pull incentives for antibacterial medicines. Health Aff (Millwood) 40:1758–1765. 10.1377/hlthaff.2021.00688. 34724432

[26] Årdal C, Røttingen J-A, Opalska A, Van Hengel AJ, Larsen J. 2017. Pull incentives for antibacterial drug development: an analysis by the transatlantic task force on antimicrobial resistance. Clin Infect Dis 65:1378–1382. 10.1093/cid/cix526. 29017240

[27] Mahase E. 2020. UK launches subscription style model for antibiotics to encourage new development. BMJ 369:m2468. 10.1136/bmj.m2468. 32554429

[28] Rex JH, Outterson K. 2020. UK antibiotic subscription pilot implies pull incentive of up to $4b across the G20. https://amr.solutions/2020/03/29/uk-antibiotic-subscription-pilot-implies-pull-incentive-of-up-to-4b-across-the-g20/.

[29] Rex JH, Krause KM. 2021. 21st century cures V2.0 — PASTEUR act is embedded! https://amr.solutions/2021/06/22/21st-century-cures-v2-0-pasteur-act-is-embedded/.

[30] Vorperian S, Quake S. 2021. The PASTEUR Act can help win the war against superbugs, *on* STAT News. https://www.statnews.com/2021/06/25/pasteur-act-help-fight-superbugs-antimicrobial-resistance/. STAT, Boston, MA.

[31] Czaplewski L, Bax R, Clokie M, Dawson M, Fairhead H, Fischetti VA, Foster S, Gilmore BF, Hancock REW, Harper D, Henderson IR, Hilpert K, Jones BV, Kadioglu A, Knowles D, Ólafsdóttir S, Payne D, Projan S, Shaunak S, Silverman J, Thomas CM, Trust TJ, Warn P, Rex JH. 2016. Alternatives to antibiotics—a pipeline portfolio review. Lancet Infect Dis 16:239–251. 10.1016/S1473-3099(15)00466-1. 26795692

[32] Theuretzbacher U, Piddock LJV. 2019. Non-traditional antibacterial therapeutic options and challenges. Cell Host Microbe 26:61–72. 10.1016/j.chom.2019.06.004. 31295426

[33] Rex JH, Fernandez Lynch H, Cohen IG, Darrow JJ, Outterson K. 2019. Designing development programs for non-traditional antibacterial agents. Nat Commun 10:3416. 10.1038/s41467-019-11303-9. 31366924 PMC6668399

[34] Greig SL. 2016. Obiltoxaximab: first global approval. Drugs 76:823–830. 10.1007/s40265-016-0577-0. 27085536

[35] Giacobbe DR, Dettori S, Di Bella S, Vena A, Granata G, Luzzati R, Petrosillo N, Bassetti M. 2020. Bezlotoxumab for preventing recurrent *Clostridioides difficile* infection: a narrative review from pathophysiology to clinical studies. Infect Dis Ther 9:481–494. 10.1007/s40121-020-00314-5. 32632582 PMC7452994

[36] Tsai C-W, Morris S. 2015. Approval of raxibacumab for the treatment of inhalation anthrax under the US Food and Drug Administration “Animal Rule”. Front Microbiol 6:1320. 10.3389/fmicb.2015.01320. 26648915 PMC4664625

[37] Xu W, Ohanjanian L, Sun J, Cui X, Suffredini D, Li Y, Welsh J, Eichacker PQ. 2017. A systematic review and meta-analysis of preclinical trials testing anti-toxin therapies for *B. anthracis* infection: a need for more robust study designs and results. PLoS One 12:e0182879. 10.1371/journal.pone.0182879. 29200425 PMC5714336

[38] Lienhardt C, Raviglione MC. 2020. TB elimination requires discovery and development of transformational agents. Appl Sci 10:2605. 10.3390/app10072605.

[39] Wu M-L, Aziz DB, Dartois V, Dick T. 2018. NTM drug discovery: status, gaps and the way forward. Drug Discov Today 23:1502–1519. 10.1016/j.drudis.2018.04.001. 29635026 PMC6078814

[40] Paulin S, Alm RA, Beyer P. 2020. A novel pre-clinical antibacterial pipeline database. PLoS One 15:e0236604. 10.1371/journal.pone.0236604. 32722726 PMC7386594

[41] Butler MS, Paterson DL. 2020. Antibiotics in the clinical pipeline in October 2019. J Antibiot (Tokyo) 73:329–364. 10.1038/s41429-020-0291-8. 32152527 PMC7223789

[42] Theuretzbacher U, Gottwalt S, Beyer P, Butler M, Czaplewski L, Lienhardt C, Moja L, Paul M, Paulin S, Rex JH, Silver LL, Spigelman M, Thwaites GE, Paccaud J-P, Harbarth S. 2019. Analysis of the clinical antibacterial and antituberculosis pipeline. Lancet Infect Dis 19:e40–e50. 10.1016/S1473-3099(18)30513-9. 30337260

[43] Theuretzbacher U, Bush K, Harbarth S, Paul M, Rex JH, Tacconelli E, Thwaites GE. 2020. Critical analysis of antibacterial agents in clinical development. Nat Rev Microbiol 18:286–298. 10.1038/s41579-020-0340-0. 32152509

[44] Access to Medicine Foundation. 2020. Antimicrobial resistance benchmark 2020. Amsterdam, The Netherlands.

[45] Hoy SM. 2021. Contezolid: first approval. Drugs 81:1587–1591. 10.1007/s40265-021-01576-0. 34365606 PMC8536612

[46] Abdul-Mutakabbir JC, Alosaimy S, Morrisette T, Kebriaei R, Rybak MJ. 2020. Cefiderocol: a novel siderophore cephalosporin against multidrug-resistant Gram-negative pathogens. Pharmacotherapy 40:1228–1247. 10.1002/phar.2476. 33068441

[47] Sato T, Yamawaki K. 2019. Cefiderocol: discovery, chemistry, and *in vivo* profiles of a novel siderophore cephalosporin. Clin Infect Dis 69:S538–S543. 10.1093/cid/ciz826. 31724047 PMC6853759

[48] Zhanel GG, Deng C, Zelenitsky S, Lawrence CK, Adam HJ, Golden A, Berry L, Schweizer F, Zhanel MA, Irfan N, Bay D, Lagacé-Wiens P, Walkty A, Mandell L, Lynch JP, Karlowsky JA. 2021. Lefamulin: a novel oral and intravenous pleuromutilin for the treatment of community-acquired bacterial pneumonia. Drugs 81:233–256. 10.1007/s40265-020-01443-4. 33247830

[49] Iterum Therapeutics. 2021. Iterum therapeutics receives complete response letter from U.S. Food and Drug Administration for oral sulopenem. https://www.iterumtx.com/news/press-releases/detail/73/iterum-therapeutics-receives-complete-response-letter-from. Iterum Therapeutics, Dublin, Ireland.

[50] Sadek M, Juhas M, Poirel L, Nordmann P. 2020. Genetic features leading to reduced susceptibility to aztreonam-avibactam among metallo-β-lactamase-producing *Escherichia coli* isolates. Antimicrob Agents Chemother 64:e01659-20. 10.1128/AAC.01659-20. 32988825 PMC7674043

[51] Shields RK, Doi Y. 2020. Aztreonam combination therapy: an answer to metallo-β-lactamase–producing Gram-negative bacteria? Clin Infect Dis 71:1099–1101. 10.1093/cid/ciz1159. 31802110 PMC7428391

[52] Cotroneo N, Rubio A, Critchley IA, Pillar C, Pucci MJ. 2020. *In vitro* and *in vivo* characterization of tebipenem, an oral carbapenem. Antimicrob Agents Chemother 64:e02240-19. 10.1128/AAC.02240-19. 32423950 PMC7526814

[53] Jain A, Utley L, Parr TR, Zabawa T, Pucci MJ. 2018. Tebipenem, the first oral carbapenem antibiotic. Expert Rev Anti Infect Ther 16:513–522. 10.1080/14787210.2018.1496821. 30014729

[54] Lepak AJ, Wang W, Andes DR. 2020. Pharmacodynamic evaluation of MRX-8, a novel polymyxin, in the neutropenic mouse thigh and lung infection models against Gram-negative pathogens. Antimicrob Agents Chemother 64:e01517-20. 10.1128/AAC.01517-20. PMC7577140 32868332

[55] MicuRx. 2021. Pipeline, MRX-8. http://www.micurx.com/pipeline. MicuRx, Shanghai, China.

[56] Monash University. 2021. New antibiotic to combat deadly bacterial ‘superbugs’ enters clinical trials. https://www.monash.edu/discovery-institute/news-and-events/news/2021-articles/new-antibiotic-to-combat-deadly-bacterial-superbugs-enters-clinical-trials. Monash University, Clayton, Australia.

[57] Qpex Biopharma. 2021. Qpex Biopharma initiates phase 1 study of QPX9003 targeting antibiotic-resistant pathogens. https://www.qpexbio.com/qpexbiopharmaqpx9003. Qpex Biopharma, San Diego, CA.

[58] Sinovent. 2021. FDA approved QIDP and FTD of XNW4107. https://www.sinovent.com.cn/en/News/info.aspx?itemid=124. Sinovent, Jiangsu, People’s Republic of China.

[59] TenNor Therapeutics. 2020. TenNor initiated TNP-2198 phase 1b/IIa clinical trials. http://www.tennorx.com/en/h-nd-63.html. TenNor Therapeutics, Jiangsu, China.

[60] Working Group on New TB Drugs. 2021. Clinical pipeline. https://www.newtbdrugs.org/pipeline/clinical. The Working Group for New TB Drugs, New York, NY.

[61] Sutherland HS, Tong AST, Choi PJ, Blaser A, Franzblau SG, Cooper CB, Upton AM, Lotlikar M, Denny WA, Palmer BD. 2020. Variations in the C-unit of bedaquiline provides analogues with improved biology and pharmacology. Bioorg Med Chem 28:115213. 10.1016/j.bmc.2019.115213. 31810890

[62] Xu J, Converse PJ, Upton AM, Mdluli K, Fotouhi N, Nuermberger EL. 2021. Comparative efficacy of the novel diarylquinoline TBAJ-587 and bedaquiline against a resistant *rv0678* mutant in a mouse model of tuberculosis. Antimicrob Agents Chemother 65:e02418-20. 10.1128/AAC.02418-20. 33526488 PMC8097419

[63] Pages LB, Aguirre DB, Bates RH, Pichel JC, Provencio JE, Pethe K. 2017. Antituberculosis agent. US Patent US10.

[64] Working Group on New TB Drugs. 2021. GSK-286. https://www.newtbdrugs.org/pipeline/compound/gsk-286. The Working Group for New TB Drugs, New York, NY.

[65] François B, Mercier E, Gonzalez C, Asehnoune K, Nseir S, Fiancette M, Desachy A, Plantefève G, Meziani F, de Lame P-A, Laterre P-F, MASTER 1 study group. 2018. Safety and tolerability of a single administration of AR-301, a human monoclonal antibody, in ICU patients with severe pneumonia caused by *Staphylococcus aureus*: first-in-human trial. Intensive Care Med 44:1787–1796. 10.1007/s00134-018-5229-2. 30343314

[66] Fowler VG, Jr, Das AF, Lipka-Diamond J, Schuch R, Pomerantz R, Jáuregui-Peredo L, Bressler A, Evans D, Moran GJ, Rupp ME, Wise R, Corey GR, Zervos M, Douglas PS, Cassino C. 2020. Exebacase for patients with *Staphylococcus aureus* bloodstream infection and endocarditis. J Clin Invest 130:3750–3760. 32271718 10.1172/JCI136577 PMC7324170

[67] Bulger E, May A, Robinson BR, Evans D, Henry S, Green J, Toschlog E, Sperry J, Fagenholz P, Martin N, Dankner W, Maislin G, Wilfret D, Bernard A, ACCUTE Study Investigators. 2020. A novel immune modulator for patients with necrotizing soft tissue infections (NSTI): results of a multicenter, phase 3 randomized controlled trial of reltecimod (AB 103). Ann Surg 272:469–478. 32657946 10.1097/SLA.0000000000004102

[68] McGovern BH, Ford CB, Henn MR, Pardi DS, Khanna S, Hohmann EL, O’Brien EJ, Desjardins CA, Bernardo P, Wortman JR, Lombardo M-J, Litcofsky KD, Winkler JA, McChalicher CWJ, Li SS, Tomlinson AD, Nandakumar M, Cook DN, Pomerantz RJ, Auninš JG, Trucksis M. 2021. SER-109, an investigational microbiome drug to reduce recurrence after *Clostridioides difficile* infection: lessons learned from a phase 2 trial. Clin Infect Dis 72:2132–2140. 10.1093/cid/ciaa387. 32255488 PMC8204772

[69] Ray A, Jones C. 2016. Does the donor matter? Donor vs patient effects in the outcome of a next-generation microbiota-based drug trial for recurrent *Clostridium difficile* infection. Future Microbiol 11:611–616. 26986546 10.2217/fmb.16.10

[70] Langdon A, Schwartz DJ, Bulow C, Sun X, Hink T, Reske KA, Jones C, Burnham C-AD, Dubberke ER, Dantas G, CDCPEP. 2021. Microbiota restoration reduces antibiotic-resistant bacteria gut colonization in patients with recurrent *Clostridioides difficile* infection from the open-label PUNCH CD study. Genome Med 13:28. 33593430 10.1186/s13073-021-00843-9 PMC7888090

[71] Seres Therapeutics. 2020. Seres therapeutics announces positive topline results from SER-109 phase 3 ECOSPOR III study in recurrent *C difficile* infection. https://ir.serestherapeutics.com/news-releases/news-release-details/seres-therapeutics-announces-positive-topline-results-ser-109. Seres Therapeutics, Cambridge, MA.

[72] BiomeBank. 2021. BiomeBank submits for market authorisation of world first microbial therapy. https://www.biomebank.com/news/biome-bank-submits-for-market-authorisation-of-world-first-microbial-therapy. BiomeBank, Thebarton, Australia.

[73] Atox Bio. 2020. Atox Bio announces FDA acceptance to file the NDA for reltecimod to treat suspected organ dysfunction or failure in patients with necrotizing soft tissue infection (“flesh-eating disease”). https://www.atoxbio.com/news/. Atox Bio, Durham, NC.

[74] Francois B, Jeannet R, Daix T, Walton AH, Shotwell MS, Unsinger J, Monneret G, Rimmelé T, Blood T, Morre M, Gregoire A, Mayo GA, Blood J, Durum SK, Sherwood ER, Hotchkiss RS. 2018. Interleukin-7 restores lymphocytes in septic shock: the IRIS-7 randomized clinical trial. JCI Insight 3:e98960. 10.1172/jci.insight.98960. PMC5922293 29515037

[75] Xiong YQ, Estellés A, Li L, Abdelhady W, Gonzales R, Bayer AS, Tenorio E, Leighton A, Ryser S, Kauvar LM. 2017. A human biofilm-disrupting monoclonal antibody potentiates antibiotic efficacy in rodent models of both *Staphylococcus aureus* and *Acinetobacter baumannii* Infections. Antimicrob Agents Chemother 61:e00904-17. 10.1128/AAC.00904-17. 28717038 PMC5610488

[76] Ryser S, Tenorio E, Estellés A, Kauvar LM. 2019. Human antibody repertoire frequently includes antibodies to a bacterial biofilm associated protein. PLoS One 14:e0219256. 10.1371/journal.pone.0219256. 31287831 PMC6615618

[77] Blondiaux N, Moune M, Desroses M, Frita R, Flipo M, Mathys V, Soetaert K, Kiass M, Delorme V, Djaout K, Trebosc V, Kemmer C, Wintjens R, Wohlkönig A, Antoine R, Huot L, Hot D, Coscolla M, Feldmann J, Gagneux S, Locht C, Brodin P, Gitzinger M, Déprez B, Willand N, Baulard AR. 2017. Reversion of antibiotic resistance in *Mycobacterium tuberculosis* by spiroisoxazoline SMARt-420. Science 355:1206–1211. 10.1126/science.aag1006. 28302858

[78] Working Group on New TB Drugs. 2021. BVL-GSK098. https://www.newtbdrugs.org/pipeline/compound/bvl-gsk098. Working Group on New TB Drugs, New York, NY.

[79] Huang DB, Sader HS, Rhomberg PR, Gaukel E, Borroto-Esoda K. 2021. Anti-staphylococcal lysin, LSVT-1701, activity: in vitro susceptibility of *Staphylococcus aureus* and coagulase-negative staphylococci (CoNS) clinical isolates from around the world collected from 2002 to 2019. Diagn Microbiol Infect Dis 101:115471. 10.1016/j.diagmicrobio.2021.115471. 34280671

[80] Huang DB, Gaukel E, Kerzee N, Borroto-Esoda K, Lowry S, Xiong YQ, Abdelhady W, Bayer AS. 2021. Efficacy of antistaphylococcal lysin LSVT-1701 in combination with daptomycin in experimental left-sided infective endocarditis due to methicillin-resistant *Staphylococcus aureus*. Antimicrob Agents Chemother 65:e00508-21. 10.1128/AAC.00508-21. PMC8284455 34097491

[81] Stokes JM, Lopatkin AJ, Lobritz MA, Collins JJ. 2019. Bacterial metabolism and antibiotic efficacy. Cell Metab 30:251–259. 10.1016/j.cmet.2019.06.009. 31279676 PMC6990394

[82] Lee BS, Kalia NP, Jin XEF, Hasenoehrl EJ, Berney M, Pethe K. 2019. Inhibitors of energy metabolism interfere with antibiotic-induced death in mycobacteria. J Biol Chem 294:1936–1943. 10.1074/jbc.RA118.005732. 30530783 PMC6369303

[83] Pethe K, Bifani P, Jang J, Kang S, Park S, Ahn S, Jiricek J, Jung J, Jeon HK, Cechetto J, Christophe T, Lee H, Kempf M, Jackson M, Lenaerts AJ, Pham H, Jones V, Seo MJ, Kim YM, Seo M, Seo JJ, Park D, Ko Y, Choi I, Kim R, Kim SY, Lim SBin, Yim S-A, Nam J, Kang H, Kwon H, Oh C-T, Cho Y, Jang Y, Kim J, Chua A, Tan BH, Nanjundappa MB, Rao SPS, Barnes WS, Wintjens R, Walker JR, Alonso S, Lee S, Kim J, Oh S, Oh T, Nehrbass U, Han S-J, No Z, et al. 2013. Discovery of Q203, a potent clinical candidate for the treatment of tuberculosis. Nat Med 19:1157–1160. 10.1038/nm.3262. 23913123

[84] Urban M, Šlachtová V, Brulíková L. 2021. Small organic molecules targeting the energy metabolism of *Mycobacterium tuberculosis*. Eur J Med Chem 212:113139. 10.1016/j.ejmech.2020.113139. 33422979

[85] Zigangirova NA, Nesterenko LN, Sheremet AB, Soloveva AV, Luyksaar SI, Zayakin ES, Balunets DV, Gintsburg AL. 2021. Fluorothiazinon, a small-molecular inhibitor of T3SS, suppresses salmonella oral infection in mice. J Antibiot (Tokyo) 74:244–254. 10.1038/s41429-020-00396-w. 33479520

[86] Sarshar M, Behzadi P, Ambrosi C, Zagaglia C, Palamara AT, Scribano D. 2020. FimH and anti-adhesive therapeutics: a disarming strategy against uropathogens. Antibiotics 9:397. 10.3390/antibiotics9070397. PMC7400442 32664222

[87] Waring MJ, Arrowsmith J, Leach AR, Leeson PD, Mandrell S, Owen RM, Pairaudeau G, Pennie WD, Pickett SD, Wang J, Wallace O, Weir A. 2015. An analysis of the attrition of drug candidates from four major pharmaceutical companies. Nat Rev Drug Discov 14:475–486. 10.1038/nrd4609. 26091267

[88] Payne DJ, Gwynn MN, Holmes DJ, Pompliano DL. 2007. Drugs for bad bugs: confronting the challenges of antibacterial discovery. Nat Rev Drug Discov 6:29–40. 10.1038/nrd2201. 17159923

[89] CARB-X. 2021. CARB-X celebrates five years of progress in early-stage product development against antibiotic-resistant bacteria. https://carb-x.org/carb-x-news/carb-x-celebrates-five-years-of-progress-in-early-stage-product-development-against-antibiotic-resistant-bacteria/. CARB-X, Boston, MA.

[90] GARDP. 2021. Research & development. https://gardp.org/what-we-do/research-development/. GARDP, Geneva, Switzerland.

[91] Mullard A. 2020. Pharmaceutical firms commit US$1 billion to antibiotic development. Nat Rev Drug Discov 19:575. 10.1038/d41573-020-00143-8. 32788679

[92] Grzelak EM, Choules MP, Gao W, Cai G, Wan B, Wang Y, McAlpine JB, Cheng J, Jin Y, Lee H, Suh J-W, Pauli GF, Franzblau SG, Jaki BU, Cho S. 2019. Strategies in anti-*Mycobacterium tuberculosis* drug discovery based on phenotypic screening. J Antibiot (Tokyo) 72:719–728. 10.1038/s41429-019-0205-9. 31292530 PMC6760628

[93] Lienhardt C, Nunn A, Chaisson R, Vernon AA, Zignol M, Nahid P, Delaporte E, Kasaeva T. 2020. Advances in clinical trial design: weaving tomorrow’s TB treatments. PLoS Med 17:e1003059. 10.1371/journal.pmed.1003059. 32106220 PMC7046183

[94] Duffy EM, Buurman ET, Chiang SL, Cohen NR, Uria-Nickelsen M, Alm RA. 2021. The CARB-X portfolio of nontraditional antibacterial products. ACS Infect Dis 7:2043–2049. 10.1021/acsinfecdis.1c00331. 34346202

[95] Alm RA, Gallant K. 2020. Innovation in antimicrobial resistance: the CARB-X perspective. ACS Infect Dis 6:1317–1322. 10.1021/acsinfecdis.0c00026. 32202756

[96] ND4BB ENABLE. 2021. European Gram Negative AntiBacterial Engine ENABLE. http://nd4bb-enable.eu/enable-portfolio. ND4BB ENABLE, Brussels, Belgium.

[97] Rajavel M, Kumar V, Nguyen H, Wyatt J, Marshall SH, Papp-Wallace KM, Deshpande P, Bhavsar S, Yeole R, Bhagwat S, Patel M, Bonomo RA, Fvd A, Wright GD. 2021. Structural characterization of diazabicyclooctane β-lactam “enhancers” in complex with penicillin-binding proteins PBP2 and PBP3 of *Pseudomonas aeruginosa*. mBio 12:e03058-20. 10.1128/mBio.03058-20. 33593978 PMC8545096

[98] Barnes MD, Kumar V, Bethel CR, Moussa SH, O’Donnell J, Rutter JD, Good CE, Hujer KM, Hujer AM, Marshall SH, Kreiswirth BN, Richter SS, Rather PN, Jacobs MR, Papp-Wallace KM, van den Akker F, Bonomo RA. 2019. Targeting multidrug-resistant *Acinetobacter* spp.: sulbactam and the diazabicyclooctenone β-lactamase inhibitor ETX2514 as a novel therapeutic agent. mBio 10:e00159-19. 10.1128/mBio.00159-19. 30862744 PMC6414696

[99] Barnes MD, Taracila MA, Good CE, Bajaksouzian S, Rojas LJ, van Duin D, Kreiswirth BN, Jacobs MR, Haldimann A, Papp-Wallace KM, Bonomo RA. 2019. Nacubactam enhances meropenem activity against carbapenem-resistant *Klebsiella pneumoniae* producing KPC. Antimicrob Agents Chemother 63:e00432-19. 10.1128/AAC.00432-19. 31182530 PMC6658744

[100] Peptilogics. 2021. First-in-human data show PLG0206 intravenously administered was well tolerated in healthy volunteers, with linear PK over the dose range. https://www.peptilogics.com/news/peptilogics-presents-positive-first-in-human-phase-1-data-for-its-lead-engineered-antibacterial-peptide-plg0206-at-idweek-2021/. Peptilogics, Pittsburgh, PA.

