# Supplementary material for: Analysis of the Clinical Pipeline of Treatments for Drug-Resistant Bacterial Infections: Despite Progress, More Action Is Needed
Source: Antimicrob Agents Chemother. 2022 Mar 15;66(3):e01991-21. doi: 10.1128/aac.01991-21 (PMC8923189; doi:10.1128/aac.01991-21)
Supplement: Supplemental file 1 — Supplemental text, Fig. S1 to S3, and Table S1. Download aac.01991-21-s0001.pdf, PDF file, 0.2 MB [file aac.01991-21-s0001.pdf]

Supplemental Information

Analysis of the clinical pipeline of treatments for drug resistant bacterial infections: despite progress, more action is needed

Mark S. Butler,[a,b] Valeria Gigante,[c] Hatim Sati,[c] Sarah Paulin,[c] Laila Al-Sulaiman,[c] John H. Rex,[d,e] Prabhavathi Fernandes,[f,g] Cesar A. Arias,[h,i] Mical Paul,[j,k] Guy E. Thwaites,[l,m] Lloyd Czaplewski,[n] Richard A. Alm,[o] Christian Lienhardt,[p] Melvin Spigelman,[q] Lynn L. Silver,[r] Norio Ohmagari,[s] Roman Kozlov,[t] Stephan Harbarth,[u,v] Peter Beyer[c]

[a] MSBChem Consulting, Brisbane, Queensland, Australia
[b] Institute for Molecular Bioscience, University of Queensland, Australia
[c] Antimicrobial Resistance Division, WHO, Geneva, Switzerland
[d] F2G Limited, Eccles, Cheshire, United Kingdom
[e] McGovern Medical School at The University of Texas Health Science Center at Houston, Houston, Texas, USA
[f] Scientific Advisory Committee, GARDP, Geneva, Switzerland
[g] The National Biodefense Science Board, Health and Human Services, Washington DC, USA
[h] Center for Infectious Diseases Research, Houston Methodist Research Institute, Houston, Texas, USA
[i] Center for Infectious Diseases, UTHealth School of Public Health, Houston, Texas, USA
[j] Infectious Diseases Institute, Rambam Health Care Campus, Haifa, Israel
[k] The Ruth and Bruce Rappaport Faculty of Medicine, Technion-Israel Institute of Technology, Haifa, Israel
[l] Oxford University Clinical Research Unitgrid.412433.3, Ho Chi Minh City, Vietnam.
[m] Centre for Tropical Medicine and Global Health, Nuffield Department of Clinical Medicine, Oxford University, Oxford, UK
[n] Chemical Biology Ventures Ltd, Abingdon, Oxfordshire, UK
[o] CARB-X, Boston University, Boston, Massachusetts, USA
[p] Université de Montpellier, Institut de Recherche pour le Développement, INSERM—Montpellier, France
[q] TB Alliance, New York, New York, USA
[r] LL Silver Consulting, New Jersey, USA
[s] National Center for Global Health and Medicine, Tokyo, Japan
[t] Institute of Antimicrobial Chemotherapy, Smolensk State Medical University, Smolensk, Russia.
[u] National Center for Infection Prevention, Swissnoso, Bern, Switzerland
[v] Infection Control Programme, Geneva University Hospitals and Faculty of Medicine, WHO Collaborating Center for Patient Safety, Geneva, Switzerland.

This contribution has been prepared strictly in a personal capacity and reflects the view of the authors. The views expressed must not be attributed to the WHO, its Secretariat, or its Member States.

Address correspondence to Mark S. Butler, mark@msbchem.com, and Peter Beyer, beyerp@who.int

Overview of the drug development process. Before clinical trials can start, an Investigational New Drug Application (IND) must be submitted and approved by the US Food and Drug Administration (FDA), European Medicines Agency (EMA), Japanese Pharmaceuticals and Medical Devices Agency (PMDA), Chinese National Medical Products Administration (NMPA), CDSCO (Central Drugs Standard Control Organization of the Government of India), Australian Therapeutic Goods Administration (TGA) or equivalent national agency. The antibacterial candidates are then evaluated in a Phase 1 trial or trials to identify the maximum dose that can be administered safely without causing severe side effects. Next, Phase 2 trials are undertaken to further evaluate the dosing regimen and obtain clinical efficacy data. Finally, Phase 3 trial or trials are used to compare the safety and effectiveness of the new antibacterial agents against the current standard treatment. Upon successful completion of Phase 3, a New Drug Application (NDA: FDA, PMDA, NMPA and CDSCO), or a Marketing Authorization Application (MAA: EMA and TGA) must be submitted to a Regulatory Agency, who then decide whether the antibacterial drug is approved for treating patients.

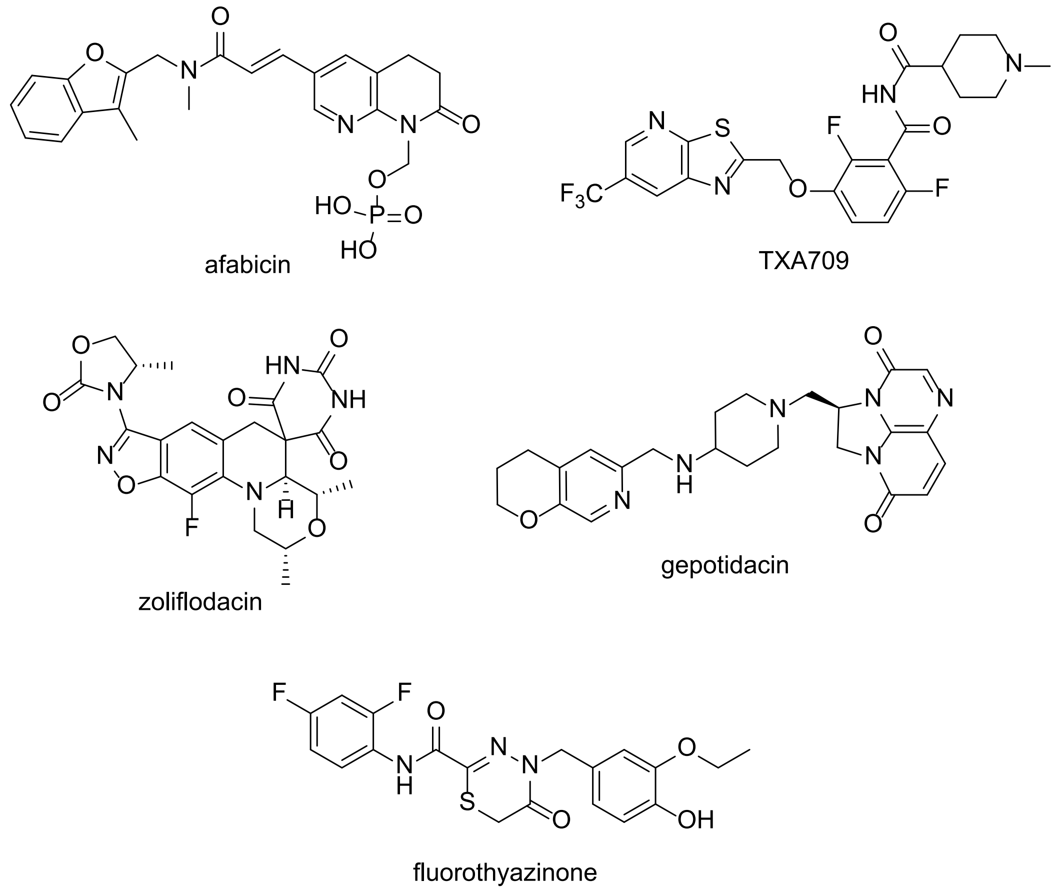

FIG S1 Structures of compounds with new pharmacophores being developed to treat WHO Priority Pathogens infections

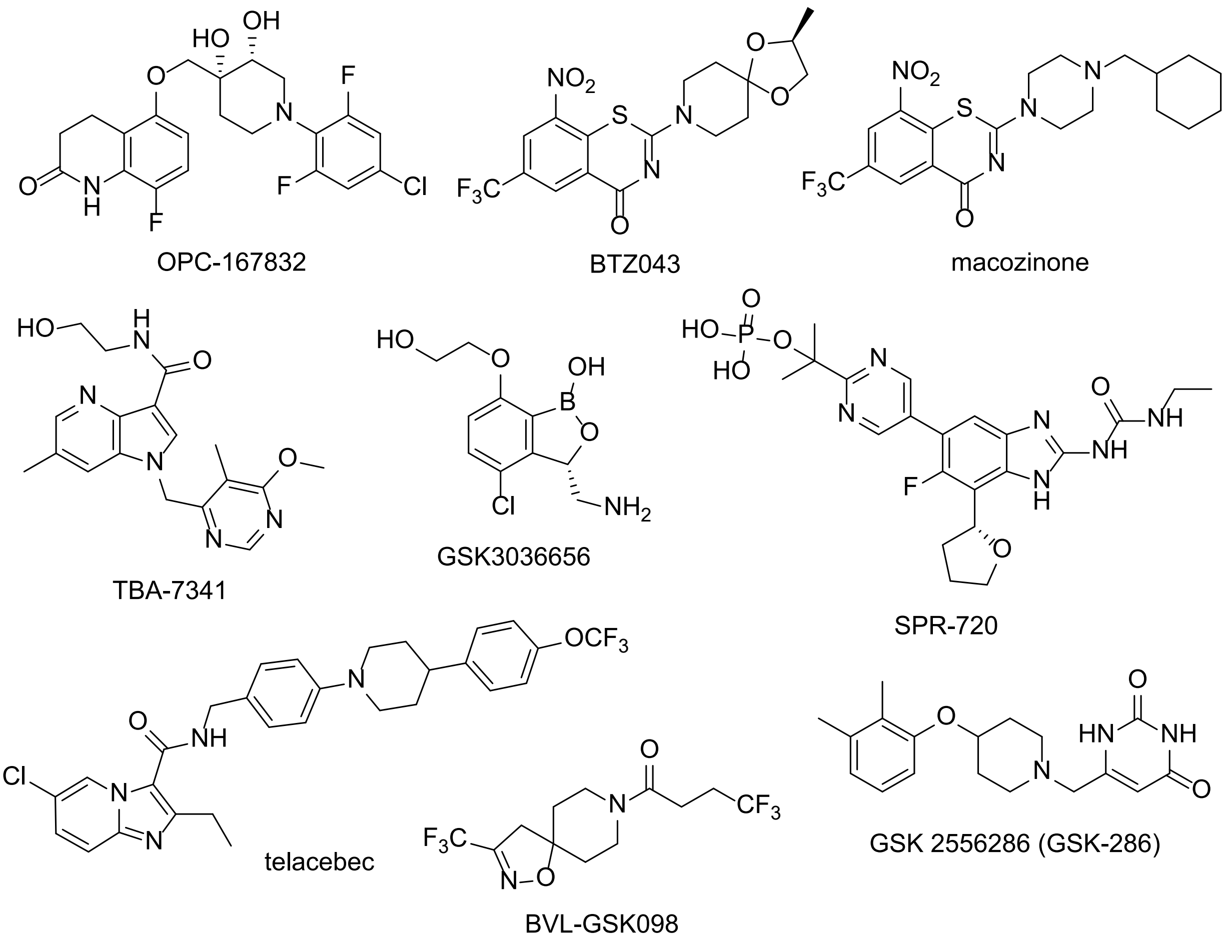

FIG S2 Structures of compounds with new pharmacophores being developed to treat TB and NTM infections

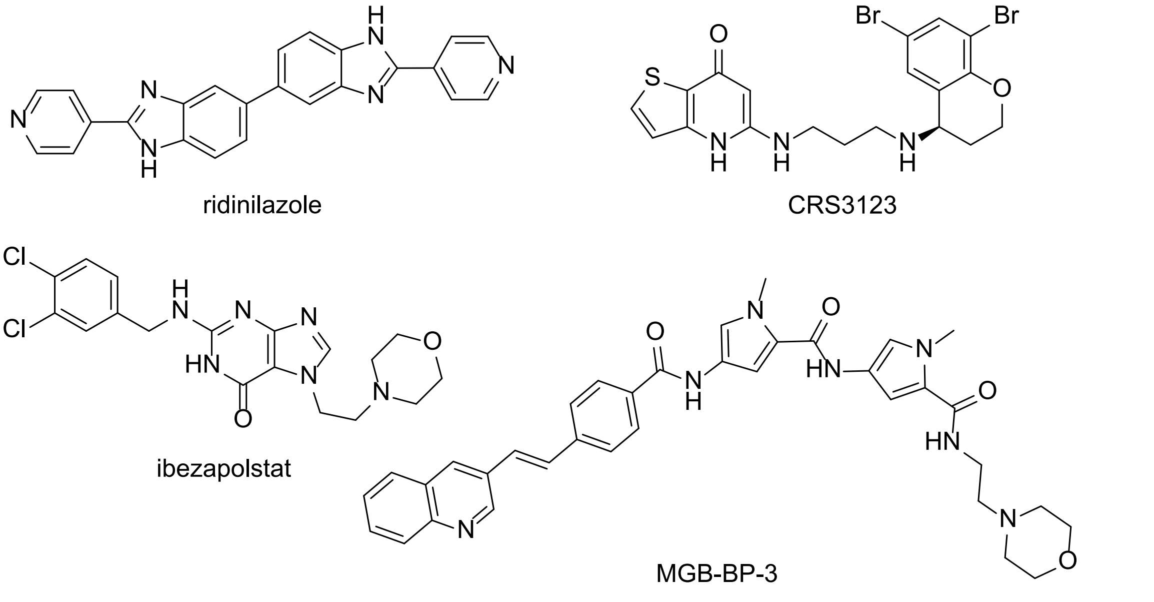

FIG S3 Structures of compounds with new pharmacophores being developed to treat *C. difficile* infections

TABLE S1 Agents not under active development due to a public termination announcement, removal from a company's development pipeline, or no clinical development update for over three years with new traditional antibacterial pharmacophores underlined

Name (synonym)	Phase	Antibiotic class	Pathogen activity	Developer	Year	Notes and/or reasons for discontinuation
GSK-3342830	1	Siderophore-cephalosporin	Gram-ve	GSK	2017	Adverse event
AIC-499 + unknown BLI	1	β-Lactam + BLI	Gram-ve	AiCuris	2017	Unknown
DS-2969	1	New class (GyrB inhibitor)	*C. difficile*	Daiichi Sankyo	2017	Adverse event
514G3 (omodenbamab)[a]	1/2	Anti-*S. aureus* IgG monoclonal antibody	*S. aureus*	Xbiotech	2017	Study completed in Feb 2017
SQ-109	2/3	Ethambutol derivative[b]	TB	Sequella	2017	Unknown
SPR-741+ β-lactam	1	Polymyxin (potentiator) + β-lactam	Gram-ve	Spero/Everest Medicines	2018	Business decision; moving forward with SPR206
Cefilavancin (TD-1792, RD-1792)	3	Glycopeptide-cephalosporin hybrid	*S. aureus*	R Pharm/Theravance	2018	Unknown; not in R-Pharm pipeline
Ramoplanin	2	Lipodepsipeptide	*C. difficile*	Ology Bioservices	2018	Discontinued in Feb 2018
Ancremonam (BOS-228, LYS-228)	2	Monobactam	CRE	Boston Pharmaceuticals	2018	Licensed in Oct 2018, but did not move into clinical trials
Cadazolid	3	Oxazolidinone-quinolone hybrid	*C. difficile*	Actelion Pharmaceuticals	2019	Discontinued in Mar 2018; Actelion acquired by J&J
RC-01 (T 1228)	1	New class (LpxC inhibitor)	Gram-ve	Recida/FUJIFILM Toyama	2019	Safety
GT-1	1	Siderophore-cephalosporin	Gram-ve	Geom	2019	Unknown
MK-3866	1	BLI	Gram-ve	Merck	2019	Business and program changes
Murepavadin (POL7080)[c]	3	Peptide	*P. aeruginosa*	Polyphor	2019	See Footnote 3
AR-105 (aerucin)[a]	2	Anti-*P. aeruginosa* fully human IgG1 mAb	*P. aeruginosa*	Aridis (Serum Institute of India)	2019	Trial completed in Apr 2019, but not on pipelines
BCM-0184	1	Not disclosed (likely peptide)	*S. aureus*	Biocidium	2019	Trial not registered and no update
Iclaprim	3	Trimethoprim	*S. aureus*	Motif Bio	2020	Company ceased operations in Mar 2020
MEDI-3902 (gremubamab)	2	Anti-*P. aeruginosa* IgG mAb	*S. aureus*	AstraZeneca (MedImmune)	2020	Safety/efficacy reasons
OPS-2071	2	Quinolone	*C. difficile*	Otsuka	2020	Development strategy
TP-271[a]	1	Tetracycline	*S. aureus* and *S. pneumoniae*	La Jolla (Tetraphase)	2021	Tetraphase acquired in Jul 2020, La Jolla looking to license
TP-6076[a]	1	Tetracycline	*A. baumannii*	La Jolla (Tetraphase)	2021	Tetraphase acquired in Jul 2020, La Jolla looking to license

[a] These antibacterials were previously listed as 'in development' in the 2020 WHO Pipeline Report. [b] Although SQ-109 is structurally derived from ethambutol, SQ109 has different modes of action that includes inhibition of mMpl3, which is a trehalose monomycolate transporter important in cell wall synthesis (1). [c] The development IV administered murepavadin was discontinued in 2019, but clinical trials are planned to treat *P. aeruginosa* infections in people with cystic fibrosis using inhaled administration.

Reference: (1) Li W, et al. 2014. Novel insights into the mechanism of inhibition of MmpL3, a target of multiple pharmacophores in *Mycobacterium tuberculosis*. Antimicrob Agents Chemother 58:6413−6423.
